# A unique genetic variation with respect to blast (*Pyricularia oryzae* Cavara) resistance in rice (*Oryza sativa* L.) varieties in Vietnam

**DOI:** 10.1270/jsbbs.22073

**Published:** 2023-04-25

**Authors:** Ngoc B. Nguyen, Nguyet T. M. Nguyen, Nhai T. Nguyen, Linh H. Le, Nghia T. La, Thuy T. T. Nguyen, Mary Jeany Yanoria, Nagao Hayashi, Hiroki Saito, Mitsuhiro Obara, Tadashi Sato, Yoshimichi Fukuta

**Affiliations:** 1 Agricultural Genetics Institute, Km 2, Pham Van Dong, Bac Tu Liem, Hanoi, Vietnam; 2 Plant Resources Center (PRC), An Khanh, Hoai Duc, Hanoi, Vietnam; 3 Ministry of Agriculture and Rural Development, No2 Ngoc Ha, Ba Dinh, Hanoi, Vietnam; 4 International Rice Research Institute (IRRI), Los Baños Laguna, The Philippines; 5 Institute of Agrobiological Sciences (NIAS), National Agricultural and Food Research Organization (NARO), Tsukuba, Ibaraki 305-8602, Japan; 6 Tropical Agriculture Research Front (TARF), Japan International Research Center for Agricultural Sciences (JIRCAS), Ishigaki, Okinawa 907-0002, Japan; 7 Japan International Research Center for Agricultural Sciences, 1-1 Ohwashi, Tsukuba, Ibaraki 305-8686, Japan; 8 Graduate School of Agricultural Science, Tohoku University, 468-1 Aramaki Aza Aoba, Aoba-ku, Sendai, Miyagi 980-8572, Japan

**Keywords:** resistance, genetic variation, differential system, blast disease, rice (*Oryza sativa* L.), Vietnam

## Abstract

A unique genetic variation with respect to blast resistance was clarified in 201 rice accessions from Vietnam. These accessions were classified into three clusters—A, B1, and B2—based on their reactions to 26 standard differential blast isolates selected in Vietnam. Cluster A was the dominant cultivar group in Vietnam and the most susceptible of the three clusters. Cluster B1 was the smallest group and the most resistant. Cluster B2 was the second-most dominant group and of intermediate resistance between clusters A and B1. The percentages of accessions comprising each cluster varied by region and area. Accessions in cluster A were distributed widely throughout Vietnam and had the highest frequencies in both the Central and North regions. Accessions in cluster B2 were found with highest frequencies in the mountainous and intermediate areas of the North region. Accessions in cluster B1 were found with highest frequencies in the Central region and Red River Delta area (North region). These results suggest that rice accessions in Vietnam were basically susceptible (cluster A) or of intermediate resistance (cluster B2), and that high-resistance cultivars were mainly distributed in the low altitude areas, such as the Red River Delta area and Central region.

## Introduction

Vietnam has over 4,000 years of experience cultivating rice (*Oryza sativa* L.) and is considered a global center of rice variation. The vast genetic diversity of its rice arises from its geographical situation, latitudinal range, differences in habitats, and diversity of ecosystems. Most rice germplasm in Vietnam comprises Indica Group, Japonica Group, and wild rice relatives that are considered the traditional, native rice landraces (Khanh *et al.* 2021). However, most landraces and germplasm have long growth duration and low yield and are only sporadically planted by ethnic minorities in mountainous and remote areas. Since improved rice cultivars were introduced in Vietnam after green revolution with the release of improved rice cultivars carrying the semi-dwarf gene, rice yields have significantly increased, allowing both national food security and export (Duong and Thanh 2019). However, those cultivars were introduced from nearby countries such as Thailand and China and from the International Rice Research Institute (IRRI), and their genetic backgrounds of rice cultivars introduced in Vietnam have been therefore poorly understood (Nguyen *et al.* 2015). Thus, the necessity of clarification of the genetic variation in rice germplasm has remained in Vietnam.

Rice blast caused by the fungal pathogen *Pyricularia oryzae* Cavara (syn. *Magnaporthe oryzae* B.C. Couch) is one of the major factors limiting the production of rice in the world (Bonman 1992) because the pathogen can infect and cause lesions on almost all organs of rice plants (Le *et al.* 2010). Using cultivars that are resistant to blast is the most ecofriendly and economical approach to managing the disease. However, these cultivars often become susceptible within a few years after their release as populations of virulent blast races that can overcome the resistance become more prevalent (Koizumi 2007, Zeigler *et al.* 1995). In Vietnam, in 2012, the total area affected by rice blast disease was 366,412 ha, of which 11,400 ha was affected by severe infections and eight ha was fully lost; reduction in milling yield was about 10% to 25% (Statistical Yearbook of Vietnam 2013).

The interaction between host resistance and blast fungus virulence can be explained by the gene-for-gene theory: every resistance gene in the host corresponds to avirulence gene in the pathogen (Flor 1971, Silue *et al.* 1992). Based on the gene-for-gene theory, differential rice varieties (DVs) harboring blast resistance genes have been developed for studying the pathogenicity of blast fungus. The most efficient set of DVs is the set of monogenic lines (MLs) developed by the *IRRI–Japan Collaborative Research Project* and donated to the International Rice Research Institute (IRRI) by the Ministry of Agriculture, Forestry and Fisheries of Japan (Tsunematsu *et al.* 2000). These MLs were intended to cover 24 resistance genes—*Pish*, *Pia*, *Pib*, *Pit*, *Pii*, *Pi3*, *Pi5*(t), *Pik-s*, *Pik-m*, *Pi1*, *Pik-h*, *Pik*, *Pik-p*, *Pi7*(t), *Pi9*(t), *Piz*, *Piz-5*, *Piz-t*, *Pita-2* (donor cultivar: Pi No. 4), *Pita-2* (Reiho), *Pi11*(t), *Pi12*(t), *Pita* (K1), *Pita* (CP1), *Pi19*(t), and *Pi20*(t)—by backcrosses between donor cultivars and the Chinese susceptible Japonica Group rice cultivar ‘Lijiangxintuanheigu’ (LTH) as the recurrent parent. However, the number of backcrosses was one to three times only, and the morphological traits and heading dates varied among the MLs. Moreover, IRRI–Japan collaborative research project later found that *Pi11*(t) was not actually included (unpublished data). Telebanco-Yanoria *et al.* (2010) developed near-isogeneic lines (NILs) with LTH genetic background containing 11 resistance genes—*Pib*, *Pia*, *Pi3*, *Pik-s*, *Pik-h*, *Pik*, *Pi1*, *Pi9*(t), *Piz-5*, *Pita-2*, and *Pita*—as advanced DVs based on six times backcrossing with LTH as the recurrent parent. These NILs all had morphological traits and heading dates similar to those of LTH.

The MLs and LTH NILs have been used widely as international DVs owing to their high ability to differentiate races of blast fungus from tropical to temperate areas: Cambodia (Fukuta *et al.* 2014), West Africa (Odjo *et al.* 2014), south central coast of Vietnam (Nguyen *et al.* 2015), Yunnan, China (Li *et al.* 2016), Japan (Kawasaki-Tanaka *et al.* 2016), Kenya (Fukuta *et al.* 2019a), Northern and Central Vietnam (Nguyen *et al.* 2020), Mekong River Delta, Vietnam (Fukuta *et al.* 2020), Laos (Xangsayasane *et al.* 2020), and Indonesia (Kadeawi *et al.* 2021). Specifically, the differential systems consist of DVs and standard differential blast isolates (SDBIs) that were developed based on pathological analyses from West Africa (Odjo *et al.* 2016), Bangladesh (Khan *et al.* 2016), Vietnam (Nguyen *et al.* 2020), Laos (Xangsayasane *et al.* 2020), and Indonesia (Kadeawi *et al.* 2021), and have been used in studies on pathogenicity of blast fungus and resistance genes in rice cultivars. The SDBIs in these differential systems have been used to clarify genetic variations among rice germplasm and to characterize blast resistance gene(s) in materials from several Asian countries (Telebanco-Yanoria *et al.* 2008), Japan (Kawasaki-Tanaka and Fukuta 2014), Bangladesh (Khan *et al.* 2017), West Africa (Odjo *et al.* 2017), northern Laos (Muto *et al.* 2019), Kenya (Fukuta *et al.* 2019b), Ethiopia (Taddesse *et al.* 2020), and Cambodia (Orn *et al.* 2020), and in one landrace, Ingngoppor-tinawon, from the Philippines (Mizobuchi *et al.* 2014).

There are few genetic studies on blast resistance gene(s) in rice cultivars of Vietnam. Inukai *et al.* (1995) found 4 resistance genes, *Pi1*, *Pik-h*, *Pita*, and an unknown gene in the genetic background of the Vietnamese landrace ‘Tetep’, which was known to be broadly resistant to blast disease. Thuan *et al.* (2003) found a resistance gene *Pi-VT7* in the Vietnamese landrace ‘Chiembac’ in the same region as *Pita* and *Pi12*(t) on chromosome 12. Quynh and Bong (1999) found 23 highly resistant cultivars and 80 moderately resistant cultivars from the screening of 500 accessions from the Mekong River Delta, South Vietnam. Khuyen *et al.* (2011) investigated the reactions of 341 rice landraces to three blast isolates and recorded a wide variation of blast resistance among them; however, the back data, such as the geographical origins of the landraces, the ecosystems of rice cultivation, and categories of Indica or Japonica Groups, were not considered.

Under the Japan International Research Center for Agricultural Sciences (JIRCAS) research project “Blast Research Network for Stable Rice Production” from 2006 to 2020, a common set of 79 simple sequence repeat (SSR) markers (McCouch *et al.* 2002) was used to clarify genetic variation in chromosome components among rice accessions and classify them according to each country or region, as follows. Kawasaki-Tanaka and Fukuta (2014) used 64 of the 79 SSR markers to classify Japanese Japonica Group rice accessions into two clusters of irrigated lowland and upland cultivars. Wunna *et al.* (2016) used 65 SSR markers to divide rice accessions from Myanmar into Indica and Japonica Groups. Odjo *et al.* (2017) used 61 SSR markers to classify accessions from West Africa into three cluster groups of Japonica Group including upland accessions, Indica Group including lowland accessions, and *O. glaberrima* and *O. barthii*. Fukuta *et al.* (2019b) used 67 SSR markers to classify accessions from Kenya into two cluster groups of Japonica Group and Indica Group. Muto *et al.* (2019) used 67 SSR markers to classify accessions from northern Laos into thrww cluster groups of lowland Japonica Group, upland Japonica Group, and Indica Group. Orn *et al.* (2020) used 64 SSR markers to classify accessions from Cambodia into two cluster groups of Japonica Group and Indica Group. Taddesse *et al.* (2020) used 50 SSR markers to classify Ethiopian rice into Indica Group and Japonica Group. Thus, the polymorphism data of SSR markers can be used to classify rice accessions into categories (Indica and Japonica Groups), ecosystems for rice cultivations (lowland and upland), and rice species.

Studies on genetic variation for blast resistance in Vietnamese rice germplasm and genetic analyses of resistance gene(s) in Vietnamese rice cultivars are quite limited, making it difficult to understand the genetic situation and variation of rice cultivars in Vietnam. In this study, we tried to clarify the genetic variations in rice germplasm based on their resistance to SDBIs selected by Nguyen *et al.* (2020) and based on polymorphism data of SSR markers in terms of the relationships of geographical distributions of the places and ecosystems that the accessions were originally cultivated in Vietnam.

## Materials and Methods

### Rice accessions

This study examined 209 rice accessions collected in Vietnam. Accessions were from the North region (mountainous area, 17 accessions; intermediate area, 41 accessions; and the Red River Delta, 79 accessions), Central region (45 accessions), and the South region (2 accessions) (Table 1, Supplemental Table 1). Also included were another 25 accessions that were either cultivated widely across both irrigated lowland and rainfed lowland ecosystems or for which information on geographical origin was not collected. These accessions are conserved in the Plant Resources Center (PRC) of Vietnam and were provided to us by the PRC for this study. Information on whether each accession was of the Japonica or Indica Group was also shared: 57 accessions were Japonica (27.3%) and 142 were Indica (67.9%); the remaining 10 accessions (4.8%) had no information recorded for these categories. The ecosystems for rice cultivation were identified based on the topographical information of the locations at which the rice accessions were collected: 28 accessions were from irrigated lowland ecosystems, 160 were from rainfed lowland ecosystems, and 21 were from upland ecosystems. More Japonica Group accessions were from upland ecosystems (38.1%) than from irrigated lowland ecosystems (28.6%) or rainfed lowland ecosystems (25.6%).

### Classification of rice accessions using polymorphism data of SSR markers

The set of 79 SSR markers (McCouch *et al.* 2002) that were used commonly for rice classification under the JIRCAS research project “Blast Research Network for Stable Rice Production” were used to classify the rice accessions, based on differences in rice genome chromosome components (Supplemental Table 2). These were selected from a public database (https://www.gramene.org). The polymorphism data of 14 SSR markers could be used in this study because the other markers lacked the polymorphism data in several rice accessions.

Genomic DNA was extracted from a young leaf in each rice accession. Leaf tissue was ground in 100 μL of 0.25 N NaOH with zirconium beads in 2.0 mL tubes. A volume of 400 μL of 100 mM Tris-HCl (pH 7.5) was added and the sample was then well mixed and centrifuged for 10 minutes at 10,000 rpm. The supernatant was transferred into 1.5 mL autoclaved tubes.

PCR was performed in a 10 μL PCR mixture containing 1 μL sterile H_2_O, 1.5 μL of forward (2 μM) and reverse (2 μM) primers of SSR marker, 7.5 μL of 2× Quick Taq TM HS DyeMix (Toyobo Co., Ltd., Tokyo, Japan), and 5 μL DNA concentrated to around 5–10 ng/μL. PCR amplification was carried out with the following profile: 94°C for 2 minutes, followed by 40 cycles of 30 seconds at 94°C, 30 seconds at 55°C, and 1 minute at 68°C. PCR products were electrophoresed on 2% agarose gels in 1× TAE buffer at 150 V for 90–120 minutes and DNA fragments were visualized with ethidium bromide. The polymorphism data of each accession was recorded based on the banding pattern and compared with those of control cultivars Nipponbare and Kasalath. The numbers of alleles were counted as different polymorphisms in each SSR marker among rice accessions.

### Inoculation with blast isolates, and estimation of resistance

Putative resistance genes were determined in 201 of the 209 rice accessions at the Agricultural Genetic Institute (AGI), Vietnam, by using a set of 25 monogenic lines (Tsunematsu *et al.* 2000) or LTH NILs (Telebanco-Yanoria *et al.* 2010), together harboring 23 resistance genes, as DVs and two susceptible controls (the Indica Group line ‘US-2’ [Fukuta *et al.* 2022] and the Chinese Japonica Group cultivar ‘Lijiangxintuanheigu’ [LTH]), (Supplemental Table 1). Nipponbare and Kasalath were used as controls for Japonica Group and Indica Group cultivars, respectively.

A total of 26 SDBIs from Vietnam (Nguyen *et al.* 2020) were used to evaluate the variation of blast resistance among rice accessions. The evaluations were done with three seedlings from each accession with two replications. The seeds were sown in a plastic cell tray (14 × 32 cells; cells 16 mm diameter, 25 mm deep) and then placed in a greenhouse at 25°C for 2 weeks. Two-week-old seedlings were inoculated with each of the SDBIs at a volume of 80 mL with the spore concentration standardized to 30–50 × 10^4^ spores/mL. The tray of plants was placed on a swivel chair and rotated while being misted with the spore suspension until droplets were visible. The inoculated plants were put in an incubator room at 25°C with high relative humidity (>90%) for 20 hours, and then transferred to a greenhouse with temperature of 25°C and 60% relative humidity for 1 week. Infection was evaluated at seven days after inoculation. The degree of infection of each seedling was scored based on a scale of 0 to 5 as described by Hayashi and Fukuta (2009).

The resistance genes in each rice accession were postulated based on their patterns of reaction to SDBIs in comparison to the patterns of reaction of the 25 DVs.

Genetic variations of rice accessions classified based on polymorphism data of SSR markers and patterns of reaction to SDBIs were computed with Ward’s hierarchical clustering method in JMP software (SAS Institute, Inc., Cary, NC, USA).

## Results

### Classification of rice accessions based on polymorphism data of SSR markers

A total of 201 of the 209 accessions, plus two control cultivars, Nipponbare (Japonica Group) and Kasalath (Indica Group), were used for the polymorphism analysis. The 79 SSR markers showed 336 alleles, and the average number of alleles per SSR marker was 4.3 (Supplemental Table 2). The 201 accessions plus two controls were classified into three cluster groups, Ia, Ib, and II, based on the polymorphism data of 65 alleles to 14 SSR markers which were collected from all accessions (Supplemental Fig. 1, Supplemental Table 1). Cluster Ia comprised 22 accessions plus Nipponbare, and cluster Ib comprised seven accessions plus Kasalath. Cluster II comprised 172 accessions including such as Indica Group varieties, Bac Thom 7 and BC 15, and Jasmine. Polymorphism data for the remaining eight accessions were not collected.

### Relationships between cluster groups by SSR markers and rice categories

Of the 209 rice accessions, the PRC had categorized 142 accessions into Indica Group’s varieties and 57 accessions into Japonica Group’s varieties. The other ten accessions had no information recorded for these categories.

Among the rice accessions categorized as Indica Group varieties, 127 accessions (89.4%) fell into cluster II, nine (6.3%) fell into cluster Ia, and two (1.4%) fell into cluster Ib (Table 2). These results indicate that cluster II corresponded to the Indica Group.

Among the 57 accessions categorized as Japonica Group cultivars, ten accessions (17.5%) fell into cluster Ia, five (8.8%) fell into cluster Ib, and 38 (66.7%) fell into cluster II. The percentages of accessions that fell into cluster Ia and Ib were higher than those of the Indica Group. These results indicate that clusters Ia and Ib were corresponded to Japonica Group. Many rice accessions categorized as Japonica Group by the PRC included those of cluster II, and the classification might be wrong. The numbers of alleles (65) from polymorphisms of 14 SSR markers were limited, and these might be not enough for the detail classifications. The ten accessions that did not have any information recorded for category, were classified into cluster Ia (three accessions) and cluster II (seven accessions).

### Geographical distributions of groups clustered by SSR markers

For all 209 rice accessions, 10.5% fell into cluster Ia, 3.3% fell into cluster Ib, 82.3% fell into cluster II, and the remaining eight accessions accounted for 3.8% (Table 3).

In all areas and regions, rice accessions of cluster II were cultivated dominantly, and different types of rice, such as those in clusters Ia and Ib, were distributed more in the intermediate and mountainous areas in the North region. Accessions from the Red River Delta area in the North region showed a distribution of frequencies across cluster groups similar with that of the whole set of accessions. In contrast, among accessions from the intermediate and mountainous areas of the North region, although accessions in cluster II still dominated, frequencies in cluster groups Ia and Ib tended to be higher than in other areas. In particular, the frequency of cluster Ia (14.6%) in the intermediate area and the frequencies of clusters Ia and Ib (both 17.6%) in the mountainous area were the highest among all 3 areas of the North region and the Central region. In the Central region, rice accessions of cluster II were dominant (40 accessions, 88.9%), and of the remaining accessions, four accessions (8.9%) were categorized into cluster Ia and one accession (2.2%) was categorized into cluster Ib. The two accessions from the South region both fell into cluster II.

### Classification of rice accessions based on resistance to blast

We classified 201 of the 209 rice accessions, two representative varieties (Nipponbare and Kasalath), and two susceptible controls (LTH and US-2) into three cluster groups, A, B1, and B2, based on their patterns of reaction to the 26 SDBIs (Supplemental Table 1, Supplemental Fig. 2). Resistance was not evaluated in the remaining 8 accessions (3.8%). Cluster group A included 104 accessions (49.8%), cluster group B1 included 37 accessions (17.7%), and cluster group B2 included 60 accessions (28.7%) (Table 1). The susceptible controls (LTH and US-2) and Kasalath were categorized into cluster A; Nipponbare was categorized into cluster B2.

**Fig. 1.**
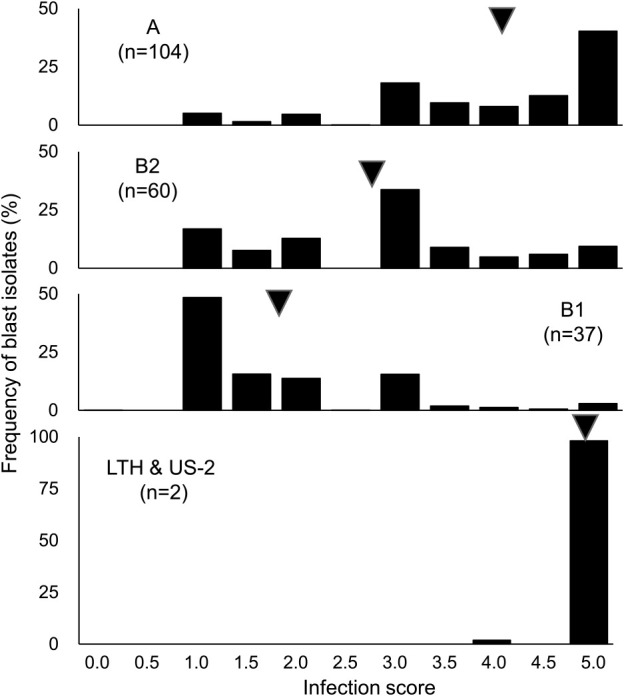
Reaction of rice accessions in Vietnam to standard differential blast isolates. Variations in each cluster group is indicated by the frequencies of infection scores to 26 standard differential blast isolates from Vietnam. Nipponbare and Kasalath were included in clusters B2 and A, respectively. ▼: Mean of infection scores.

The infection scores of the two susceptible controls LTH and US-2 with respect to the 26 SDBIs varied from 4.0 (reaction to only one SDBI) to 5.0, and the mean was 5.0 (Fig. 1). In cluster A, scores varied from 1.0 to 5.0 and the mean was 4.0. In cluster B1, infection scores ranged from 0.0 to 5.0 with a mean of 1.7, and in cluster B2, scores ranged from 1.0 to 5.0 with a mean of 2.8. These results indicated that cluster A was susceptible and cluster B1 was resistant. Cluster B2 was of intermediate resistance between clusters A and B1.

### Estimation of blast resistance genes in rice accessions

Resistance genes in the genetic background of the rice accessions were estimated based on the patterns of reaction to SDBIs in Vietnam as compared with the patterns of reaction to the set of international DVs (Supplemental Table 1).

**Fig. 2.**
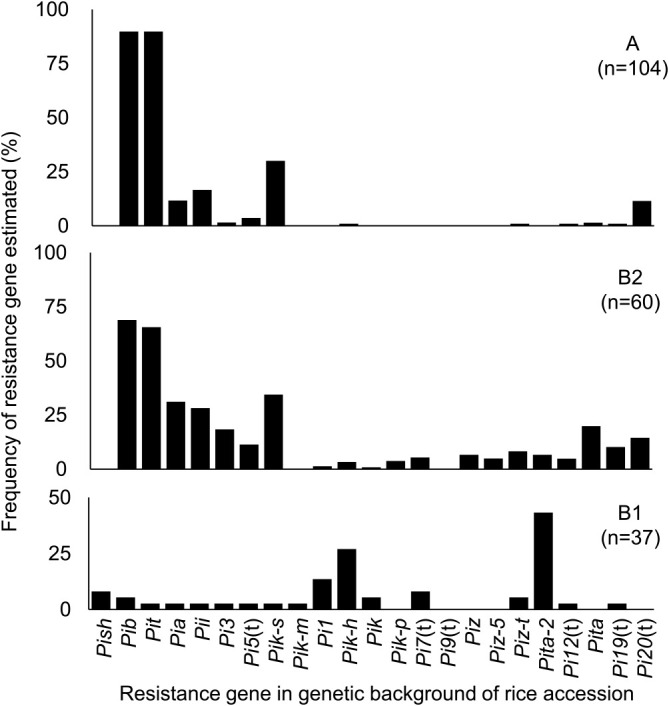
Resistance genes estimated in the genetic backgrounds of rice accessions in each cluster group. Gene estimation was based on the patterns of reaction to 26 SDBIs from Vietnam as compared with those of set of international DVs. In cluster B1, 18 rice accessions showed resistance to all SDBIs, and the resistance gene(s) could not be estimated in their genetic backgrounds.

Almost all rice accessions in cluster group A were estimated to harbor resistance genes *Pib* and *Pit* in their genetic backgrounds: the frequencies were 89.8% for each (Fig. 2). Four resistance genes, *Pia*, *Pii*, *Pik-s*, and *Pi20*(t), were also estimated to be harbored in 11.5% to 30.1% of these accessions. *Pib* and *Pit* conferred resistance to only one SDBI each: I95 (U43-i7-k177-z06-ta021) and I165 (U23-i3-k102-z01-ta032), respectively. *Pia* conferred resistance to three SDBIs: I185 (U71-i4-k175-z02-ta702), I186 (U71-i4-k176-z04-ta722), and I140 (U61-i5-k102-z04-ta423). Pii conferred resistance to six SDBIs: I62 (U63-i6-k100-z05-ta413), I185 (U17-i4-k175-z02-ta702), I106 (U63-i6-k100-z05-ta403). I186 (U71-i4-k176-z04-ta722), I201 (U63-i0-k175-z00-ta702) and I175 (U63-i0-k126-z05-ta623). *Pik-s* conferred resistance to one SDBI: I221 (U63-i7-k003-z12-ta433). *Pi20*(t) conferred resistance to three SDBIs: I95 (U43-i7-k177-z06-ta021), I143(U63-i3-k104-z01-ta421), and I123 (U63-i7-k106-z01-ta431). Thus, the main resistance genes in cluster A conferred resistance to a narrow spectrum of the 26 SDBIs. The other 17 resistance genes—*Pish*, *Pi3*, *Pi5*(t), *Pik-m*, *Pi1*, *Pik-h*, *Pik*, *Pik-p*, *Pi7*(t), *Pi9*(t), *Piz*, *Piz-5*, *Piz-t*, *Pita-2*, *Pi12*(t), *Pita*, and *Pi19*(t)—were not estimated or were found in only a small percentage of accessions (<4%).

In cluster B2, 6 estimated resistance genes—*Pib*, *Pit*, *Pia*, *Pii*, *Pik-s*, and *Pi20*(t)—were the same as in cluster A. The percentages of accessions harboring *Pib* (68.9%) and *Pit* (56.6%) were lower than in cluster A, and the frequencies of the other 4 resistance genes were increased (14.4%–34.4%). In addition to those genes, the percentages of accessions estimated to be harboring *Pi3*, *Pi5*(t), *Pik-h*, *Pik*, *Pik-p*, *Pi7*(t), *Piz*, *Piz-5*, *Piz-t*, *Pita-2*, *Pi12*(t), *Pita*, and *Pi19*(t) were also increased (3.3%–19.8%). These resistance genes conferred resistance to a wider spectrum of the 26 SDBIs (5 to 22) compared with the six resistance genes in cluster A.

In cluster B1, only three resistance genes—*Pi1*, *Pik-h* and *Pita-2*—were estimated with high frequencies, 13.5%, 25.7%, and 35.1%, respectively. The other resistance genes were not estimated or were found in only a small percentage of accessions (<8.1%). However, among the 37 accessions in cluster B1, 18 accessions (48.6%) were resistant to all SDBIs, and for these we were not able to estimate the resistance gene(s). These results indicated that they harbored unknown gene(s) or accumulated several genes in their genetic backgrounds which conferred resistance to a wide spectrum of these SDBIs and whose genetic mechanisms were different from that of clusters A and B2.

### Distributions of cluster groups for blast resistance among geographical regions and ecosystems for rice cultivation

**Fig. 3.**
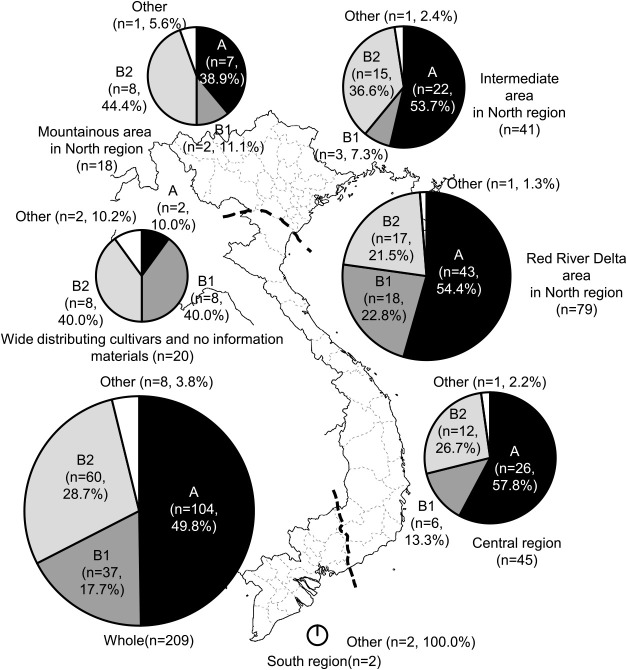
Distributions of cluster groups for blast resistance of rice accessions in four regions of Vietnam.

Cluster A was the most dominant group in the intermediate, and Red River Delta areas in the North region and in the Central region, with accessions in this cluster accounting for 53.7% to 57.8% of accessions in each area. The lowest value (38.9%) was found in the mountainous area of the North region (Fig. 3). The frequencies of accessions in cluster B2 in the mountainous and intermediate areas in the North region (36.6%–44.4%) were remarkably higher than those of the Red River Delta area in the North region and the Central region (21.5%–26.7%) and all accessions together (28.7%). In contrast, the frequencies of accessions in cluster B1 in the mountainous and intermediate areas in the North region (7.3%–11.1%) were lower than those of the other two regions (13.3%–22.8%) and all accessions together (17.7%). In regions categorized as “other”, which included widely distributed cultivars and those for which there was no information on geographical origin recorded, quite a high percentage of accessions fell into cluster B1 and cluster B2 (40.0% in each) compared with corresponding clusters in the three areas in the North and the Central regions, and for all accessions together.

With respect to ecosystems for rice cultivation, the cluster groups with highest frequencies for blast resistance compared with those for all accessions together, were found in two combinations: cluster B1 in irrigated lowland sites and cluster B2 on upland sites (Table 1). Frequencies on rainfed lowland sites were similar with those for all accessions together.

## Discussion

### Variation in blast resistance in Vietnamese rice accessions

A total of 209 rice accessions from Vietnam were analyzed to assess genetic variation with respect to their resistance to blast. We inoculated 201 accessions with 26 SDBIs (Nguyen *et al.* 2020) to clarify the genetic variations in their resistance to blast, and then those accessions were classified into cluster groups A, B1, and B2 based on their patterns of reaction to the SDBIs (Supplemental Fig. 2). Cluster A included 104 accessions (49.8%), cluster B1 included 37 accessions (17.7%), and cluster B2 included 60 accessions (28.7%). The dominant cluster (cluster A) was the group most susceptible to blast among the 3 clusters, and accessions in this group were distributed widely and with high frequency in all 3 areas of the North region (mountainous area, intermediate area, and Red River Delta) and the Central region (Figs. 1, 3). In contrast, the frequencies of the intermediate resistant group, cluster B2, varied by area and region. The mountainous and intermediate areas in the North region showed higher frequencies of accessions in cluster B2 than were found in the Red River Delta area in North region and the Central region. In contrast, the frequencies of accessions in cluster B1 were highest in the Red River Delta area and Central region, and among the other 8 accessions that were grown widely in several provinces or for which there was no information with respect to geographical origin. These results suggest that clusters A and B2 were the basic types characterizing blast resistance in Vietnam. In other words, rice cultivars in Vietnam are basically susceptible, such as those in cluster A, or of moderate resistance, such as those in cluster B2.

The resistance gene(s) of rice accessions were estimated based on the patterns of reaction to 26 SDBIs in comparison with the patterns of reaction of the set of International DVs (Supplemental Table 1, Fig. 2). Almost all rice accessions of cluster A harbor *Pib* and *Pit* in their genetic backgrounds, with four other genes, *Pia*, *Pii*, *Pik-s*, and *Pi20*(t), combined additionally in several accessions. These resistance genes correspond to narrow spectrum of the 26 SDBIs and resistance reactions were limited to a few isolates. Among the rice accessions of cluster B2, in addition to the six genes also found in cluster A, 3.3% to 19.8% of accessions were estimated to harbor an additional 12 resistance genes in their genetic backgrounds: *Pi3*, *Pi5*(t), *Pik-h*, *Pik*, *Pik-p*, *Piz*, *Piz-5*, *Piz-t*, *Pita-2*, *Pi12*(t), *Pita*, and *Pi19*(t). These resistance genes showed a wider spectrum of resistance reactions compared with that of the six resistance genes in cluster A. These results indicate that cluster B2 might be derived from the cluster A, in an adaptive response to the high stress imposed by blast disease in higher altitudes, such as the mountainous and intermediate areas in the North region.

The most resistant group, cluster B1, included only 37 accessions (17.7% of all accessions) and it was a minor type in comparison with clusters A and B2. *Pik-h* and *Pita-2* were estimated as the major resistance genes in this cluster (Fig. 3). Among the accessions in cluster B1, 18 (48.6%) were resistant to all SDBIs, so we were not able to estimate the genotype(s) of the resistance gene(s). These accessions might harbor unknown gene(s) or might have accumulated several genes conferring a wide spectrum of resistance to these SDBIs, with genetic mechanisms different from those of clusters A and B2. High frequencies of accessions in cluster B1 were found in the Red River Delta area in the North region, the Central region, and in “other” regions that included accessions grown over a wide area. These results indicate that cluster B1, which was characterized as a high-resistance group, was dominantly used in low-altitude fields, such as the Red River Delta area in the North region and in the Central region. The frequencies of accessions in cluster B1 were higher in irrigated lowland sites and frequencies of accessions in cluster B2 were higher in upland sites than they were for all accessions together.

Thus, the dominant rice accessions with respect to blast resistance changed according to geographical location. Particularly, the highest resistance varieties were most dominantly found in low altitude areas in irrigated lowlands, and the moderate-resistance group, cluster B2, was most dominant in upland areas and rainfed lowlands. These results suggested that rice accessions in cluster A might harbor the basic genotypes of resistance genes in Vietnam, and those of cluster B1 were modified from cluster A, adding the other resistance genes to increase of adaptabilities in the conditions of high-altitude area. These of cluster B1 might be introduced newly into lowland area.

This is the first report to study the genetic variations with respect to blast resistance among rice accessions from Vietnam. However, only two rice accessions from the South region were used. These were categorized into Japonica Group by the PRC and were classified into cluster II by polymorphism data of SSR markers in this study. However, their blast resistances were not able to be investigated. To fully understand the genetic variation of blast resistance throughout the whole of Vietnam, more materials from the South region, including these two accessions, will need to be evaluated. We found 18 rice accessions in cluster B1 that showed resistance to all 26 SDBIs. These were unique and might be useful gene sources for breeding blast resistance into rice of Vietnam; however, their genetic mechanisms will first need to be clarified to understand their effects in more detail.

### Relationship between resistance in rice and blast races

Nguyen *et al.* (2020) clarified the pathogenicity of 239 blast isolates from the North and Central regions of Vietnam, and showed that there were low frequencies of blast isolates virulent to (DVs for *Pish*, *Pik-m*, *Pi1*, *Pik-h*, *Pik*, *Pik-p*, *Pi7*(t), *Pi9*(t), *Piz-5*, *Pita-2*, and *Pita*, and high frequencies of blast isolates virulent to DVs for *Pib*, *Pit*, *Pia*, *Pii*, *Pi3*, *Pi5*(t), *Pik-s*, *Piz*, *Piz-t*, *Pi12*(t), *Pi19*(t), and *Pi20*(t). These results suggest that rice cultivars in Vietnam harbor resistance genes such as *Pib*, *Pit*, *Pia*, *Pii*, *Pi3*, *Pi5*(t), *Pik-s*, *Piz*, *Piz-t*, *Pi12*(t), *Pi19*(t), and *Pi20*(t) in their genetic backgrounds, based on the gene-for-gene theory (Flor 1971, Silue *et al.* 1992). Most rice accessions in clusters A and B2 harbored six resistance genes in common—*Pib*, *Pit*, *Pia*, *Pii*(t), *Pik-s*, and *Pi20*(t)—and additionally many accessions in accessions of cluster B2 included *Pi3*, *Pi5*(t), *Pik-h*, *Pik*, *Pik-p*, *Piz*, *Piz-5*, *Piz-t*, *Pita-2*, *Pi12*(t), *Pita*, and *Pi19*(t). *Pik-h* and *Pita-2* were the dominant genes in cluster B1. The frequencies of virulent blast isolates in Nguyen *et al.* (2020) corresponded well with the estimated resistance genes in rice accessions, except for *Pik*, *Pik-p*, *Piz-5*, and *Pita*. However, these virulent blast isolates for four resistance genes were found with high frequencies in the Northwest area of the North region of Vietnam (Nguyen *et al.* 2020). Rice accessions in cluster B2 including these four genes were dominantly distributed in the mountainous and intermediate areas in the North region in this study. The Northwest area in Nguyen *et al.* (2020) is included in the mountainous area in the North region in this study. Thus, all resistance genes estimated in this study corresponded with the results of virulent blast isolates found by Nguyen *et al.* (2020).

This study demonstrated well the relationships between resistance gene(s) in rice cultivars and virulence gene(s) in blast fungus postulated by the gene-for-gene theory. In other words, outbreaks of high-virulence blast races occur according to the high resistance of rice cultivars in Vietnam.

### Relationships between cluster groups by polymorphism of SSR markers and categories assigned by the PRC

Rice accessions were classified into three cluster groups, Ia, Ib, and II, based on the polymorphism data of SSR markers (Supplemental Fig. 1). Almost all rice accessions (89.4%) that were categorized by the PRC as being of the Indica Group fell into cluster II, and the remaining 6.3% and 1.4% fell into clusters Ia and Ib, respectively. These results indicated that cluster II corresponded to the Indica Group.

Accessions categorized by the PRC as being of the Japonica Group were divided into cluster Ia (17.5%), cluster Ib (8.8%), and cluster II (66.7%). Nipponbare and Kasalath were classified into clusters Ia and Ib, respectively. A total of 79 SSR markers showed 336 alleles and the average number of alleles per SSR marker was 4.3. Kawasaki-Tanaka and Fukuta (2014) classified 324 Japanese Japonica Group rice accessions into two clusters of irrigated lowland and upland cultivars by using 176 alleles of 64 SSR markers. Odjo *et al.* (2017) classified 195 rice accessions from West Africa into three cluster groups—Japonica Group including upland accessions, Indica Group including lowland accessions, and *O. glaberrima* and *O. barthii*—by using 133 alleles of 61 SSR markers. Fukuta *et al.* (2019b) classified 47 rice accessions from Kenya into two cluster groups—Japonica Group accessions and Indica Group accessions including one *O. glaberrima*—by using 202 alleles of 67 SSR markers. Muto *et al.* (2019) classified 314 accessions from northern Laos into three clusters—lowland Japonica Group, upland Japonica Group, and Indica Group—by using 234 alleles of 67 SSR markers. Orn *et al.* (2020) classified 179 accessions into two clusters—Japonica and Indica Groups—by using 252 alleles of 64 SSR markers. Each of those studies used the same set of SSR markers that we used in this study, and the average number of alleles per SSR marker in those studies varied from 2.2 to 3.9. Thus, the average number of alleles in the accessions from Vietnam (4.3) was higher than that in accessions from the other regions, which suggests that genetic variations in the genomic chromosome components were greater in rice accessions from Vietnam than in accessions from the other countries, and that those of the Japonica Group accessions categorized by PRC were complex. The high diversity of rice accessions revealed in this study reflects the fact that Vietnam is a center for rice variation with a vast genetic diversity and long history of rice cultivation (Khanh *et al.* 2021).

An Indica Group variety, Kasalath, was categorized into cluster Ib of Japonica Group (Supplemental Fig. 1). Compared with other studies, this study used fewer numbers of SSR markers and the chromosome regions analyzed were limited, and high diversity among rice accessions was found in Vietnam. These conditions might influence the classification. Further studies employing a larger number of SSR markers will be needed to confirm the variation in genomic chromosome components among rice accessions.

## Author Contribution Statement

Drs. T. T. T. Nguyen and Y. Fukuta planted and conducted this research and prepared manuscript. Mr. N. B. Nguyen and Dr. N. T. M. Nguyen carried out mainly the research works, and Ms. N. T. Nguyen and Ms. L. H. Le supported the data collection. Ms. N. T. La selected the set of rice accessions and provided the original seed from PRC. Drs. M. J. Yanoria, N. Hayashi, H. Saito, M. Obara and T. Sato attended to the field trip for the rice germplasm survey in Vietnam and to the preparation of manuscript and discussions.

## Supplementary Material

Supplemental Figures

Supplemental Tables

## Figures and Tables

**Table 1.** Relationships of rice accessions between ecosystems for rice cultivation and categories, geographical origins, cultivate groups, clusters by SSR marker and blast resistance

Category	No. of accessions (%)			
	Irrigated lowland (IL)	Rainfed lowland (RL)	Upland (UP)	Total
Regions				
Mountanous area in North region (NM)	0 (0.0)	3 (1.9)	14 (66.7)	17 (8.1)
Intermadiate area in North region (NI)	2 (7.1)	36 (22.5)	3 (14.3)	41 (19.6)
Red River Delta in North region (RRD)	13 (46.4)	84 (52.5)	1 (4.8)	98 (46.9)
Central region (C)	2 (7.1)	23 (14.4)	1 (4.8)	26 (12.4)
South region (S)	0 (0.0)	1 (0.6)	1 (4.8)	2 (1.0)
Others	11 (39.3)	13 (8.1)	1 (4.8)	25 (12.0)
Total	28 (100.0)	160 (100.0)	21 (100.0)	209 (100.0)
Cultivar groups				
Japonica Group (J)	8 (28.6)	41 (25.6)	8 (38.1)	57 (27.3)
Indica Group (I)	19 (67.9)	110 (68.8)	13 (61.9)	142 (67.9)
Others	1 (3.6)	9 (5.6)	0 (0.0)	10 (4.8)
Total	28 (100.0)	160 (100.0)	21 (100.0)	209 (100.0)
Clusters by polymorphism of SSR markers				
Ia	3 (10.7)	17 (10.6)	2 (9.5)	22 (10.5)
Ib	1 (3.6)	4 (2.5)	2 (9.5)	7 (3.3)
II	21 (75.0)	135 (84.4)	16 (76.2)	172 (82.3)
Others	3 (10.7)	4 (2.5)	1 (4.8)	8 (3.8)
Total	28 (100.0)	160 (100.0)	21 (100.0)	209 (100.0)
Clusters by blast resistance				
A	11 (39.3)	84 (52.5)	9 (42.9)	104 (49.8)
B1	8 (28.6)	26 (16.3)	3 (14.3)	37 (17.7)
B2	8 (28.6)	44 (27.3)	8 (38.1)	60 (28.7)
Others	1 (3.6)	6 (3.8)	1 (4.8)	8 (3.8)
Total	28 (100.0)	160 (100.0)	21 (100.0)	209 (100.0)

**Table 2.** Relationships between cluster groups by SSR markers and rice categories in Vietnam

Category of cultivar group in Vietnam	No. of accessions (%)				
	Cluster group based on the polymorphism data of SSR markers				
	Ia	Ib	II	Others	Total
Indica	9 (6.3)	2 (1.4)	127 (89.4)	4 (2.8)	142 (100.0)
Japonica	10 (17.5)	5 (8.8)	38 (66.7)	4 (7.0)	57 (100.0)
Others	3 (30.0)	0 (0.0)	7 (70.0)	0 (0.0)	10 (100.0)
Total	22 (10.5)	7 (3.4)	172 (82.3)	8 (3.8)	209 (100.0)

**Table 3.** Distribution of rice accessions of three cluster groups by polymorphism of SSR makers

Regions and areas		No. of accessions (%)				
		Cluster group based on the polymorphism data of SSR markers				
		Ia	Ib	II	Others	Total
North	Mountinous	3 (16.7)	3 (16.7)	11 (61.1)	1 (5.6)	18 (100.0)
	Intermediate	6 (14.6)	2 (4.9)	33 (80.5)	0 (0.0)	41 (100.0)
	Red River Delta	11 (10.9)	2 (2.0)	85 (84.2)	3 (3.0)	101 (100.0)
Central		1 (3.7)	0 (0.0)	25 (92.6)	1 (3.7)	27 (100.0)
South		0 (0.0)	0 (0.0)	2 (100.0)	0 (0.0)	2 (100.0)
Others		1 (5.0)	0 (0.0)	16 (80.0)	3 (15.0)	20 (100.0)
Total		22 (10.5)	7 (3.3)	172 (82.3)	8 (3.8)	209 (100.0)
