# Supplementary material for: A unique genetic variation with respect to blast (Pyricularia oryzae Cavara) resistance in rice (Oryza sativa L.) varieties in Vietnam
Source: Breed Sci. 2023 Apr 25;73(2):193–203. doi: 10.1270/jsbbs.22073 (PMC10316314; doi:10.1270/jsbbs.22073)
Supplement: Supplementary file 1 — Supplemental Figures [file 73_193_s1.pdf]

Nguyen et al./Breeding Science
doi: 10.1270/jsbbs.22073

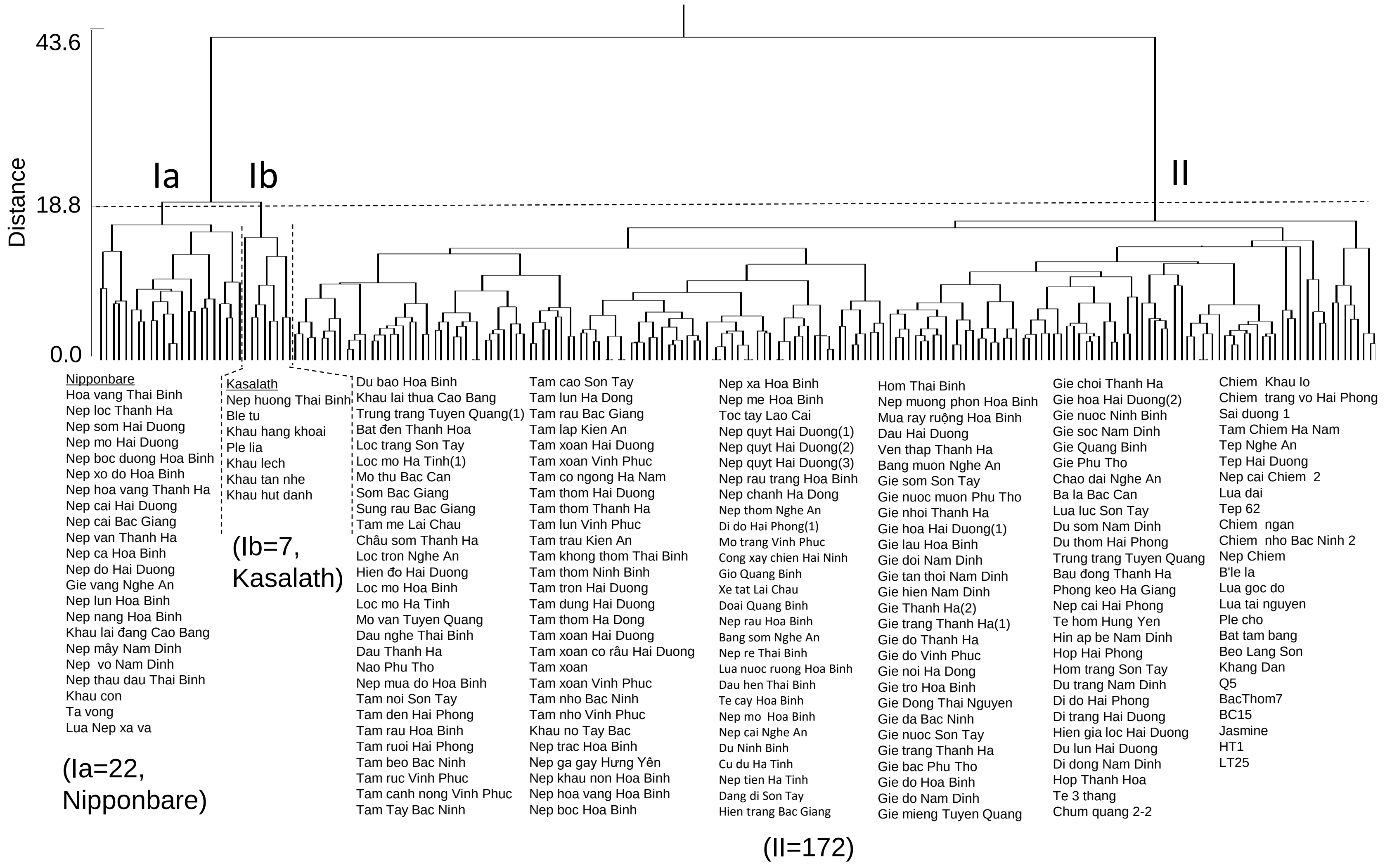

Supplemental Fig.1. Classification of rice accessions for polymorphism of DNA markers
Among 209 rice accessions, 201 rice accessions and controls, Nipponbare as Japonica Group cultivar and Kasalath as Indica Group cultivar, were used for polymorphism analysis with SSR markers. Cluster analyses were performed by Ward's hierarchical method (Ward 1963) with the software JMP version 7.02 for Windows, based on polymorphism data of 14 SSR markers among the rice accessions and controls. A total of 63 alleles data for them were used. Under line indicate rice accessions which were not used the other research works.

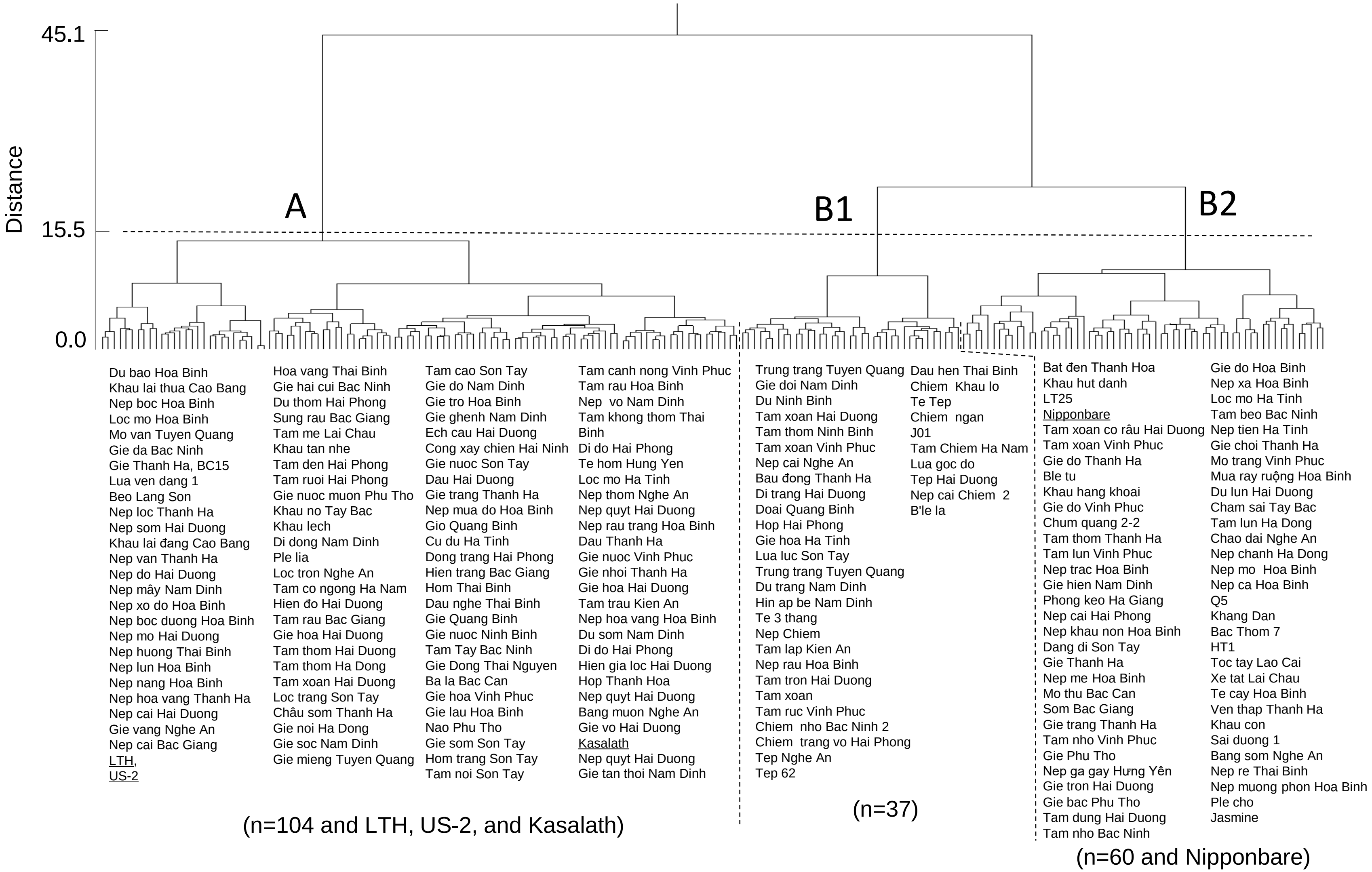

Supplemental Fig.2. Classification of rice accessions by blast resistant
Among 209 rice accessions, 201, two susceptible controls, LTH and US-2, Nipponbare as Japonica Group cultivar, and Kasalath as Indica Group cultivar, were used. Cluster analyses were performed by Ward's hierarchical method (Ward 1963) with the software JMP version 7.02 for Windows, on the basis the reactions to 26 standard differential blast isolates among the rice accessions and controls.
