# Supplementary material for: A unique genetic variation with respect to blast (Pyricularia oryzae Cavara) resistance in rice (Oryza sativa L.) varieties in Vietnam
Source: Breed Sci. 2023 Apr 25;73(2):193–203. doi: 10.1270/jsbbs.22073 (PMC10316314; doi:10.1270/jsbbs.22073)
Supplement: Supplementary file 2 — Supplemental Tables [file 73_193_s2.pdf]

Supplemental Table 1. Rice accessions used in this study

Entry No.	ID No. in Vietnam	Variety Name	Origin: Province	Origin: Region	Ecosystem for rice cultivation	Group in Vietnam	Clusters by polymorphism of SSR markers	Cluster for blast resistant	Standard differential blat isolates (Race designation by Hayashi and Fukuta. 2011)																										Estimated resistande gene(s) harboring in the genetic background
									I19 U63-i7-k177-z07-ta403	I95 U43-i7-k177-z06-ta021	I165 U23-i3-k102-z01-ta032	I62 U63-i6-k100-z05-ta413	I185 U71-i4-k175-z02-ta702	I106 U63-i6-k100-z05-ta403	I118 U63-i5-k100-z05-ta403	I143 U63-i3-k104-z01-ta421	I221 U63-i7-k003-z12-ta433	I126 U63-i7-k167-z13-ta423	I128 U63-i7-k117-z00-ta613	I127 U63-i7-k137-z17-ta403	I186 U71-i4-k176-z04-ta722	I201 U63-i0-k175-z00-ta702	I45 U63-i7-k102-z16-ta403	I175 U63-i0-k126-z05-ta623	I113 U63-i7-k100-z12-ta222	I147 U63-i7-k100-z05-ta403	I246 U63-i7-k127-z03-ta333	I2 U63-i7-k100-z05-ta602	I148 U63-i3-k100-z05-ta433	I194 U63-i7-k100-z11-ta223	I140 U61-i5-k102-z04-ta423	I75 U63-i7-k126-z07-ta413	I219 U63-i7-k110-z04-ta022	I123 U63-i7-k106-z01-ta431	
1	28	Du bao Hoa Binh	Hoa Binh	NI	RL	J	II	A	5.0	3.0	2.0	3.5	5.0	5.0	5.0	4.5	3.5	5.0	5.0	5.0	5.0	5.0	5.0	5.0	5.0	5.0	5.0	5.0	5.0	5.0	5.0	4.5	5.0	3.0	*Pib* , *Pit* , *Pik-s* , Unknown
2	29	Khau lai thua Cao Bang	Cao Bang	NM	UP	I	II	A	5.0	2.0	1.0	3.0	5.0	4.0	5.0	3.0	5.0	4.5	5.0	5.0	5.0	5.0	5.0	5.0	5.0	5.0	5.0	5.0	4.5	5.0	5.0	3.5	5.0	3.0	*Pib* , *Pit* , *Pita or Pi20* (t), Unknown
4	42	Hoa vang Thai Binh	Thai Binh	RRD	RL	I	Ia	A	5.0	3.0	1.0	3.0	4.5	3.0	3.5	5.0	5.0	4.5	5.0	3.5	3.0	3.0	5.0	1.5	3.0	5.0	5.0	4.5	5.0	5.0	1.5	4.5	4.5	4.5	*Pii* , *Pi12* (t) or two (*Pib* and *Pit*), unknown
6	50	Loc trang Son Tay	Ha Tay	C	RL	I	II	A	3.5	2.0	3.0	3.0	1.0	3.0	4.0	5.0	5.0	5.0	4.5	5.0	2.0	2.0	5.0	3.0	5.0	5.0	5.0	5.0	4.5	5.0	3.0	4.5	4.0	5.0	*Pib* , *Pit* , *Pia* , *Pii* , Unknown
7	51	Loc mo Ha Tinh(1)	Ha Tinh	C	RL	I	II	A	5.0	3.0	1.0	3.5	1.0	3.0	5.0	5.0	3.5	4.5	5.0	4.5	3.0	3.0	5.0	5.0	4.5	4.5	4.5	4.5	5.0	4.5	5.0	4.0	5.0	5.0	*Pib* , *Pit* , *Pik-s* , Unknown
10	57	Sung rau Bac Giang	Bac Giang	NI	RL	I	II	A	3.0	3.0	1.5	2.0	2.0	2.0	3.0	3.5	5.0	5.0	3.0	4.5	2.0	2.0	5.0	4.0	5.0	3.0	5.0	4.0	3.5	4.0	3.0	4.0	3.5	5.0	*Pib* , *Pit* , Unknown
11	59	Tam me Lai Chau	Lai Chau	NM	UP	I	II	A	3.5	3.0	1.0	2.0	2.0	4.0	3.0	3.5	5.0	5.0	4.5	4.5	3.0	2.0	5.0	5.0	5.0	3.0	5.0	4.5	4.0	4.5	4.5	4.5	4.5	4.5	*Pib* , *Pit* , Unknown
12	70	Châu som Thanh Ha	Thanh Hoa	C	RL	J	II	A	4.0	2.0	3.0	3.0	1.0	3.5	3.5	5.0	5.0	5.0	5.0	5.0	1.5	1.0	5.0	5.0	5.0	3.5	5.0	5.0	4.5	5.0	3.0	3.0	4.5	4.5	*Pia* , *Pib* , *Pit* , Unknown
13	81	Loc tron Nghe An	Nghe An	C	RL	J	II	A	3.5	2.0	2.0	3.0	2.0	5.0	3.0	5.0	5.0	4.5	2.0	4.5	3.0	2.0	5.0	3.0	5.0	4.5	4.0	5.0	3.5	5.0	3.0	3.0	4.0	5.0	*Pia* , *Pib* , *Pit* , Unknown
14	142	Hien đo Hai Duong	Hai Duong	RRD	UP	J	II	A	5.0	2.0	1.0	3.5	1.0	3.0	3.0	5.0	5.0	4.0	3.5	5.0	1.5	1.0	5.0	3.0	5.0	5.0	5.0	5.0	5.0	5.0	3.5	5.0	5.0	5.0	*Pib* , *Pit* , *Pii* or *Pi3* , Unknown
15	147	Loc mo Hoa Binh	Hoa Binh	NI	RL	I	II	A	5.0	1.0	1.5	3.0	2.0	5.0	5.0	1.5	5.0	5.0	3.0	5.0	3.5	3.0	5.0	3.5	5.0	5.0	5.0	4.5	5.0	5.0	5.0	5.0	5.0	1.0	*Pit* , *Pi20* (t), Unknown gene
17	150	Mo van Tuyen Quang	Tuyen Quang	NI	UP	J	II	A	5.0	2.0	1.0	3.0	3.0	5.0	3.5	3.0	4.5	4.5	4.5	5.0	3.5	3.5	5.0	1.5	5.0	3.0	3.0	4.5	4.5	5.0	4.5	5.0	4.5	3.5	*Pit* , *Pi20* (t), Unknown gene
19	155	Dau nghe Thai Binh	Thai Binh	RRD	RL	I	II	A	5.0	2.0	3.0	3.0	1.0	3.0	4.5	5.0	3.5	4.0	5.0	5.0	3.0	3.0	5.0	4.5	4.5	5.0	5.0	5.0	4.5	5.0	4.5	4.0	5.0	5.0	*Pib* , *Pit,* *Pik-s* , Unknown
20	156	Dau Thanh Ha	Thanh Hoa	C	RL	I	II	A	5.0	3.0	2.0	3.0	1.0	1.5	5.0	5.0	5.0	4.0	5.0	3.5	3.0	3.0	5.0	4.5	5.0	5.0	5.0	5.0	3.0	5.0	5.0	3.5	5.0	5.0	*Pib* , *Pit* , *Pii* , Unknown
21	164	Nao Phu Tho	Phu Tho	NI	RL	I	II	A	4.5	2.0	3.0	3.0	1.0	3.0	5.0	5.0	5.0	3.0	3.0	5.0	3.0	3.0	5.0	5.0	5.0	5.0	5.0	5.0	5.0	5.0	5.0	5.0	5.0	5.0	*Pib* , *Pit* , Unknown
22	198	Nep mua do Hoa Binh	Hoa Binh	NI	RL	I	II	A	4.0	1.0	3.0	2.0	1.0	3.0	5.0	5.0	4.0	3.0	3.0	3.5	3.0	2.0	5.0	2.0	5.0	4.5	4.0	5.0	5.0	5.0	5.0	5.0	3.5	5.0	*Pib* , *Pit* , *Pii* , Unknown
23	213	Tam noi Son Tay	Ha Tay	C	RL	I	II	A	5.0	3.0	3.0	3.0	1.0	3.0	5.0	5.0	5.0	4.5	4.5	4.5	3.0	3.0	5.0	3.5	5.0	5.0	5.0	5.0	5.0	4.5	5.0	4.5	5.0	5.0	*Pib* , *Pit* , *Pii* , Unknown
24	215	Tam den Hai Phong	Hai Phong	RRD	RL	I	II	A	4.0	3.0	3.0	2.0	3.0	3.0	4.5	5.0	5.0	3.5	3.0	3.5	3.0	3.5	3.0	3.0	3.0	4.5	5.0	5.0	4.5	5.0	5.0	3.0	5.0	5.0	*Pib* , *Pit* , *Pii* , Unknown
25	220	Tam rau Hoa Binh	Hoa Binh	NI	RL	I	II	A	4.0	3.0	4.5	3.0	1.0	3.5	5.0	4.5	5.0	5.0	3.5	4.5	3.5	3.0	5.0	3.5	3.5	5.0	5.0	5.0	5.0	4.5	5.0	5.0	5.0	5.0	*Pib* , *Pii* , Unknown
26	227	Tam ruoi Hai Phong	Hai Phong	RRD	RL	I	II	A	3.5	3.0	2.0	2.0	1.0	2.0	4.0	4.5	5.0	3.0	4.5	3.5	3.0	3.0	4.5	4.0	3.0	3.0	5.0	3.0	4.0	5.0	4.5	3.0	3.5	5.0	*Pib* , *Pi19* (t), Unknown
29	237	Tam canh nong Vinh Phuc	Vinh Phuc	RRD	RL	I	II	A	5.0	2.0	3.0	3.0	2.0	3.5	5.0	5.0	5.0	4.5	5.0	4.5	3.0	3.0	5.0	3.5	4.5	4.0	5.0	3.5	5.0	5.0	5.0	5.0	5.0	5.0	*Pib* , *Pit* , Unknown
30	240	Tam Tay Bac Ninh	Bac Ninh	RRD	RL	I	II	A	4.5	2.0	3.0	3.0	1.5	4.0	4.5	3.5	3.5	4.0	4.5	5.0	3.0	3.0	4.5	5.0	3.0	5.0	5.0	4.0	5.0	5.0	5.0	5.0	5.0	5.0	*Pib* , *Pit* , *Pik-s* , Unknown
31	242	Tam cao Son Tay	Hay Tay	RRD	RL	I	II	A	4.0	3.0	3.0	2.0	1.0	5.0	5.0	5.0	3.0	4.5	4.0	4.5	3.0	1.0	5.0	4.5	5.0	5.0	4.0	3.0	4.5	4.5	5.0	4.5	5.0	4.5	*Pib* , *Pit* , *Pik-s* , Unknown
33	244	Tam rau Bac Giang	Bac Giang	NI	RL	I	II	A	5.0	2.0	2.0	3.0	1.0	3.0	4.0	5.0	5.0	5.0	3.0	5.0	2.0	2.0	5.0	3.0	4.5	5.0	5.0	5.0	5.0	5.0	5.0	5.0	5.0	5.0	*Pib* , *Pit* , *Pii,* Unknown
37	286	Tam co ngong Ha Nam	Ha Nam	RRD	RL	I	II	A	5.0	2.0	1.5	3.0	3.0	5.0	4.5	5.0	4.5	4.5	3.0	3.5	3.0	3.0	4.5	2.0	4.5	5.0	5.0	3.0	3.5	5.0	3.0	3.0	4.0	5.0	*Pib* , *Pit* , *Pia,* Unknown
38	287	Tam thom Hai Duong*	Hai Duong	RRD	IL	I	II	A	5.0	3.0	1.0	2.0	1.5	5.0	5.0	5.0	5.0	5.0	3.0	4.0	3.5	3.0	5.0	2.0	5.0	4.5	5.0	5.0	5.0	5.0	3.5	5.0	5.0	5.0	*Pib* , *Pit* , *Pia* , Unknown
41	298	Tam trau Kien An	Hai Phong	RRD	RL	I	II	A	5.0	1.0	1.0	3.0	3.0	3.5	5.0	5.0	3.0	5.0	5.0	5.0	3.0	3.0	5.0	3.5	5.0	5.0	5.0	5.0	4.5	5.0	5.0	4.5	5.0	5.0	*Pib* , *Pit* , *Pik-s* , Unknown
42	301	Tam khong thom Thai Binh	Thai Binh	RRD	RL	J	II	A	5.0	2.0	4.0	3.0	1.0	5.0	5.0	5.0	4.5	5.0	5.0	5.0	5.0	3.0	5.0	5.0	5.0	5.0	5.0	5.0	5.0	5.0	5.0	5.0	5.0	5.0	*Pib* , Unknown
46	310	Tam thom Ha Dong*	Ha Tay	C	IL	I	II	A	5.0	2.0	1.5	2.0	1.0	3.0	4.5	5.0	5.0	5.0	3.0	4.5	3.0	3.0	5.0	2.0	3.5	3.0	5.0	5.0	5.0	5.0	3.0	3.5	5.0	5.0	*Pib* , *Pit* , *Pia, Pii,* Unknown
47	311	Tam xoan Hai Duong*	Hai Duong	RRD	IL	I	II	A	5.0	2.0	1.0	2.0	1.5	3.0	3.5	5.0	5.0	3.5	2.0	4.5	2.0	2.0	4.5	3.0	4.0	3.5	4.5	5.0	5.0	5.0	4.5	5.0	5.0	5.0	*Pib* , *Pit* , *Pii,* Unknown
53	320	Khau no Tay Bac	Tay Bac	NM	UP	I	II	A	3.0	3.0	2.0	1.0	3.0	3.0	3.5	3.5	4.0	3.0	1.5	5.0	3.0	1.0	3.0	2.0	4.0	4.5	4.5	5.0	3.5	5.0	3.5	5.0	5.0	5.0	*Pib* , *Pit* , *Pia,* One of three, *Pii,* *Pi3* , or *Pi5* (t), Unknown
57	325	Nep loc Thanh Ha	Thanh Hoa	C	RL	J	Ia	A	3.0	3.0	3.5	3.0	5.0	3.0	5.0	5.0	5.0	5.0	3.0	4.0	4.5	5.0	3.5	3.0	5.0	4.5	5.0	5.0	4.5	5.0	3.5	3.5	4.5	4.5	*Pib* , *Pit* , Unknown
58	327	Nep som Hai Duong	Hai Duong	RRD	RL	I	Ia	A	3.5	3.5	1.5	3.0	5.0	3.5	5.0	5.0	4.5	3.0	3.0	3.5	5.0	5.0	4.0	3.5	4.5	4.5	5.0	5.0	3.5	4.5	4.5	4.0	4.0	4.0	*Pib* , *Pit* , Unknown
59	333	Nep mo Hai Duong	Hai Duong	RRD	RL	I	Ia	A	5.0	3.0	3.5	3.0	4.5	5.0	5.0	5.0	5.0	4.5	3.5	4.0	4.0	5.0	5.0	5.0	5.0	5.0	5.0	5.0	5.0	5.0	5.0	5.0	5.0	5.0	*Pib* , *Pit* , Unknown
60	334	Nep boc duong Hoa Binh	Hoa Binh	NI	RL	J	Ia	A	5.0	3.0	1.0	2.0	5.0	4.0	5.0	5.0	3.5	4.0	1.0	3.5	2.0	5.0	5.0	4.5	5.0	5.0	5.0	5.0	5.0	5.0	5.0	3.5	5.0	5.0	*Pib* , *Pit, Pik-s,* Unknown
61	335	Nep hoa vang Hoa Binh*	Hoa Binh	NI	IL	I	II	A	5.0	1.0	1.5	3.0	3.0	3.0	5.0	5.0	4.0	5.0	3.0	5.0	5.0	3.0	5.0	4.0	5.0	5.0	4.0	5.0	5.0	5.0	5.0	4.0	5.0	5.0	*Pib* , *Pit* , Unknown
62	336	Nep boc Hoa Binh	Hoa Binh	NI	RL	I	II	A	5.0	2.0	2.0	4.0	5.0	3.5	5.0	1.0	5.0	5.0	5.0	5.0	5.0	5.0	5.0	5.0	5.0	4.5	4.5	3.5	5.0	5.0	5.0	3.0	5.0	1.0	*Pib* , *Pit* , *PI20* (t), Unknown
63	342	Nep xo do Hoa Binh	Hoa Binh	NI	RL	J	Ia	A	4.5	3.0	3.0	1.0	3.5	5.0	5.0	5.0	3.0	3.5	4.5	5.0	4.0	3.0	4.0	3.0	5.0	4.0	5.0	5.0	5.0	5.0	3.0	4.5	5.0	5.0	*Pib* , *Pit, Pik-s* . Unknown
64	346	Nep hoa vang Thanh Ha*	Thanh Hoa	C	IL	J	Ia	A	5.0	4.0	3.0	3.0	4.0	4.0	5.0	4.5	5.0	5.0	5.0	5.0	5.0	5.0	5.0	3.0	5.0	4.5	5.0	5.0	5.0	5.0	3.0	3.5	5.0	5.0	*Pit* , Unknown
65	348	Nep cai Hai Duong*	Hai Duong	RRD	IL	I	Ia	A	5.0	3.5	3.5	4.0	5.0	3.0	5.0	5.0	4.0	5.0	4.0	5.0	5.0	5.0	5.0	5.0	5.0	5.0	5.0	5.0	4.5	4.0	5.0	4.5	5.0	4.5	*Pib* , *Pit* , Unknown
66	349	Nep cai Bac Giang*	Bac Giang	NI	IL	I	Ia	A	5.0	3.0	3.0	3.0	5.0	3.0	5.0	5.0	4.5	4.0	2.0	5.0	5.0	5.0	5.0	5.0	5.0	5.0	5.0	5.0	5.0	5.0	5.0	4.0	5.0	5.0	*Pib* , *Pit* , Unknown
67	352	Nep van Thanh Ha	Thanh Hoa	C	RL	I	Ia	A	4.0	2.0	3.5	1.0	5.0	3.0	5.0	5.0	5.0	3.5	3.0	4.5	5.0	5.0	3.0	4.5	5.0	5.0	5.0	5.0	5.0	4.5	5.0	3.5	5.0	5.0	*Pib* , *Pit* , Unknown
71	357	Nep do Hai Duong	Hai Duong	RRD	RL	J	Ia	A	5.0	3.0	1.5	1.5	3.0	3.0	5.0	3.0	4.0	4.0	3.0	3.0	5.0	5.0	5.0	5.0	5.0	5.0	5.0	5.0	3.5	5.0	5.0	3.5	4.0	5.0	*Pib* , *Pit* , Unknown
72	398	Gie vang Nghe An	Nghe An	C	RL	J	Ia	A	5.0	3.5	3.0	3.0	4.5	3.0	5.0	5.0	4.0	4.5	5.0	5.0	5.0	5.0	5.0	5.0	5.0	5.0	5.0	5.0	5.0	5.0	5.0	5.0	5.0	5.0	*Pib* , *Pit* , Unknown
73	399	Nep lun Hoa Binh	Hoa Binh	NI	RL	I	Ia	A	4.5	4.0	4.0	4.0	4.0	5.0	4.5	5.0	4.5	4.0	3.0	4.5	5.0	5.0	4.5	4.5	4.0	5.0	4.0	5.0	5.0	5.0	5.0	5.0	5.0	5.0	Unknown
74	400	Nep nang Hoa Binh	Hoa Binh	NI	RL	J	Ia	A	5.0	4.5	3.0	3.0	5.0	5.0	5.0	5.0	3.0	5.0	3.5	4.5	5.0	5.0	5.0	5.0	4.0	5.0	4.5	4.5	5.0	5.0	5.0	5.0	4.5	5.0	*Pit* , *Pik-s* , Unknown

76	405	Nep quyt Hai Duong(1)	Hai Duong	RRD	RL	I	II	A	4.5	2.0	1.0	3.0	1.0	5.0	5.0	5.0	4.0	3.5	3.5	5.0	3.5	3.5	5.0	5.0	5.0	5.0	5.0	5.0	5.0	5.0	5.0	4.0	5.0	4.0	*Pib* , *Pit* , Unknown
77	406	Nep quyt Hai Duong(2)	Hai Duong	RRD	RL	I	II	A	4.0	2.0	1.0	3.0	1.0	3.0	5.0	5.0	3.5	4.5	3.0	4.5	3.0	3.0	3.0	5.0	4.0	5.0	3.0	5.0	5.0	5.0	5.0	5.0	5.0	4.0	*Pib* , *Pit* , *Pik-s* , Unknown
78	407	Nep quyt Hai Duong(3)	Hai Duong	RRD	RL	I	II	A	5.0	2.0	1.0	4.0	1.0	2.0	5.0	5.0	3.0	5.0	4.0	5.0	4.0	3.5	5.0	5.0	5.0	5.0	3.5	5.0	5.0	5.0	5.0	5.0	5.0	4.0	*Pib* , *Pit* , *Pik-s* , Unknown
79	410	Nep rau trang Hoa Binh	Hoa Binh	NI	RL	I	II	A	5.0	2.0	1.0	5.0	1.5	4.0	5.0	5.0	4.5	5.0	5.0	5.0	3.0	3.0	5.0	5.0	5.0	3.0	5.0	5.0	5.0	5.0	5.0	5.0	5.0	4.0	*Pib* , *Pit* , *Pik-s* , Unknown
80	414	Nep huong Thai Binh*	Thai Binh	RRD	IL	I	Ib	A	5.0	3.0	3.5	3.0	4.5	5.0	5.0	5.0	3.5	3.5	5.0	5.0	5.0	5.0	5.0	5.0	5.0	4.5	5.0	4.5	5.0	5.0	4.5	5.0	3.5	4.0	*Pib* , *Pit* , *Pik-s* , Unknown
82	420	Khau lai đang Cao Bang	Cao Bang	NM	UP	I	Ia	A	4.0	3.0	3.0	2.0	5.0	3.0	4.5	4.5	4.0	4.5	3.0	4.5	5.0	5.0	5.0	5.0	3.0	4.0	4.5	5.0	5.0	5.0	4.0	5.0	4.0	4.0	*Pib* , *Pit* , Unknown
83	451	Nep mây Nam Dinh	Nam Dinh	RRD	RL	J	Ia	A	3.5	3.0	3.5	2.0	4.5	5.0	3.0	4.5	3.0	3.0	4.0	3.5	5.0	5.0	5.0	5.0	5.0	4.5	4.0	5.0	3.5	5.0	4.5	3.0	4.0	4.0	*Pib* , *Pit* , *Pik-s* , Unknown
84	469	Nep vo Nam Dinh	Nam Dinh	RRD	RL	J	Ia	A	5.0	3.0	3.0	3.0	1.5	3.0	3.0	3.5	5.0	4.0	4.5	4.0	3.0	2.0	5.0	5.0	5.0	5.0	5.0	5.0	5.0	5.0	5.0	5.0	4.5	4.0	*Pib* , *Pit* , Unknown
86	473	Nep thom Nghe An*	Nghe An	C	IL	I	II	A	5.0	3.0	1.0	3.5	1.0	2.0	5.0	5.0	4.5	5.0	5.0	5.0	3.5	3.5	5.0	5.0	5.0	5.0	4.5	4.5	5.0	5.0	5.0	4.5	4.5	5.0	*Pib* , *Pit* , Unknown
87	502	Di do Hai Phong(1)	Hai Phong	RRD	RL	I	II	A	5.0	3.0	3.0	3.0	1.0	3.0	5.0	4.5	3.0	4.5	3.0	4.5	4.0	5.0	5.0	4.5	3.0	4.5	4.0	5.0	5.0	5.0	3.0	4.5	5.0	4.5	*Pib* , *Pit* , *Pik-s* , Unknown
89	514	Cong xay chien Hai Ninh	—	Other	RL	I	II	A	5.0	3.0	2.0	3.0	1.5	3.0	3.5	5.0	3.0	5.0	3.5	5.0	3.0	1.0	5.0	5.0	4.0	4.5	5.0	3.5	3.0	5.0	4.0	4.0	5.0	5.0	*Pib* , *Pit* , *Pik-s* , Unknown
90	515	Gie Quang Binh	Quang Binh	C	RL	I	II	A	4.0	1.0	3.0	3.0	1.0	3.0	4.5	4.5	3.0	3.5	3.0	3.5	2.0	1.0	5.0	3.5	4.0	5.0	5.0	4.0	3.5	5.0	4.5	3.5	4.5	3.5	*Pib* , *Pit* , *Pik-s* , Unknown
102	544	Cu du Ha Tinh	Ha Tinh	C	RL	I	II	A	5.0	1.0	3.0	3.5	1.0	3.0	3.5	4.5	4.5	5.0	3.0	4.0	3.0	1.5	3.0	4.0	3.5	5.0	3.5	4.5	4.5	4.5	3.5	3.5	5.0	5.0	*Pib* , *Pit* , *Pia* , Unknown
105	554	Hien trang Bac Giang	Bac Giang	NI	RL	I	II	A	3.0	1.0	3.0	3.0	1.0	4.5	4.5	5.0	3.5	4.5	3.5	4.5	3.0	2.0	4.5	4.0	4.0	5.0	5.0	5.0	3.5	4.5	4.5	4.0	4.5	5.0	*Pib* , *Pit* , *Pik-s* , Unknown
106	556	Hom Thai Binh	Thai Binh	RRD	RL	I	II	A	3.0	1.0	3.5	3.0	1.0	4.0	4.0	5.0	4.0	5.0	3.0	5.0	3.0	1.0	4.0	5.0	4.0	4.5	4.5	5.0	4.5	5.0	5.0	3.5	5.0	5.0	*Pib* , *Pit* , Unknown
109	578	Dau Hai Duong	Hai Duong	RRD	RL	J	II	A	3.5	3.0	3.0	2.0	1.0	3.0	5.0	5.0	3.5	5.0	3.0	4.5	3.0	1.0	5.0	5.0	5.0	3.0	5.0	5.0	3.5	5.0	5.0	3.0	4.0	5.0	*Pib* , *Pit* , *Pik-s* , Unknown
111	584	Bang muon Nghe An	Nghe An	C	RL	J	II	A	5.0	3.5	1.0	3.0	1.0	5.0	4.5	5.0	3.0	5.0	3.0	5.0	3.0	3.0	5.0	5.0	5.0	5.0	4.0	3.0	5.0	5.0	5.0	5.0	5.0	5.0	*Pib* , *Pit* , *Pik-s* , Unknown
112	587	Gie som Son Tay	Ha Tay	C	RL	I	II	A	3.5	2.0	3.5	3.0	1.0	3.0	5.0	4.5	4.5	4.5	3.0	5.0	3.0	1.0	3.5	5.0	5.0	5.0	4.5	4.5	4.0	5.0	5.0	5.0	5.0	5.0	*Pib* , *Pit* , Unknown
113	589	Gie nuoc muon Phu Tho	Phu Tho	NI	RL	J	II	A	4.5	3.0	1.5	2.0	1.0	3.5	3.5	5.0	4.0	3.5	3.0	3.5	3.0	3.0	2.0	5.0	3.0	3.5	5.0	3.0	3.0	4.5	5.0	3.0	5.0	5.0	*Pib* , *Pit* , Unknown
114	595	Gie nuoc Vinh Phuc	Vinh Phuc	RRD	RL	I	II	A	5.0	2.0	2.0	3.0	1.0	3.0	5.0	5.0	3.5	5.0	4.5	5.0	3.0	2.0	4.5	5.0	5.0	5.0	4.5	5.0	4.0	5.0	5.0	5.0	5.0	5.0	*Pib* , *Pit* , *Pii* , Unknown
116	604	Gie vo Hai Duong	Hai Duong	RRD	RL	I	II	A	5.0	3.0	1.0	3.0	1.0	3.5	3.5	5.0	3.0	4.5	3.0	5.0	3.0	3.0	5.0	5.0	5.0	3.0	3.5	5.0	3.5	4.5	4.5	4.0	3.5	4.0	*Pib* , *Pit* , *Pik-s* , Unknown
117	605	Gie Thanh Ha(1)	Thanh Hoa	C	RL	J	II	A	3.0	1.0	1.0	3.0	5.0	3.0	3.5	2.0	4.0	5.0	4.0	5.0	4.5	5.0	5.0	3.5	4.5	4.5	4.0	5.0	3.0	4.0	4.0	4.5	4.5	2.0	*Pib* , *Pit* , *Pi20* (t), Unknown
118	606	Gie hai cui Bac Ninh	Bac Ninh	RRD	RL	I	II	A	5.0	3.0	1.0	3.0	2.0	3.0	4.0	3.5	3.5	4.5	5.0	5.0	4.0	3.0	5.0	1.5	5.0	4.0	4.5	3.5	3.5	5.0	3.0	4.0	5.0	5.0	*Pib* , *Pit* , *Pi5* (t), *Pik-s* , Unknown
120	611	Gie hoa Vinh Phuc	Vinh Phuc	RRD	RL	I	II	A	4.5	2.0	3.0	3.0	3.0	3.0	4.0	5.0	3.5	5.0	4.5	5.0	3.0	3.0	5.0	3.5	4.0	3.5	4.0	5.0	5.0	5.0	4.5	3.0	4.5	5.0	*Pib* , *Pit* , *Pii* , *Pik-s* , Unknown
121	613	Gie nhoi Thanh Ha	Thanh Hoa	C	RL	I	II	A	5.0	2.0	2.0	3.0	1.5	3.0	5.0	5.0	5.0	5.0	5.0	4.5	4.0	1.5	4.5	5.0	5.0	5.0	4.5	5.0	5.0	5.0	5.0	4.5	5.0	5.0	*Pib* , *Pit* , Unknown
122	627	Gie hoa Hai Duong(1)	Hai Duong	RRD	RL	I	II	A	4.5	3.0	2.0	4.5	1.0	3.0	5.0	5.0	4.5	5.0	5.0	5.0	3.5	1.0	5.0	5.0	5.0	4.5	5.0	5.0	4.5	5.0	4.5	4.0	5.0	4.5	*Pib* , *Pit* , Unknown
123	629	Gie lau Hoa Binh	Hoa Binh	NI	RL	I	II	A	3.0	2.0	3.0	3.0	3.0	3.0	3.5	5.0	4.0	5.0	5.0	4.5	3.5	2.0	5.0	4.5	5.0	5.0	4.5	4.5	5.0	5.0	5.0	3.5	5.0	5.0	*Pib* , *Pit* , Unknown
125	640	Gie tan thoi Nam Dinh	Nam Dinh	RRD	RL	I	II	A	3.0	2.0	1.0	3.0	2.0	3.5	5.0	3.5	4.5	4.0	4.5	5.0	3.0	3.0	2.0	5.0	5.0	3.0	3.5	4.5	4.5	4.5	5.0	5.0	5.0	3.5	*Pib* , *Pit* , *Pii* , *Pi20* (t), Unknown
128	645	Gie trang Thanh Ha(1)	Thanh Hoa	C	RL	I	II	A	3.5	3.0	3.0	3.0	1.0	3.0	5.0	5.0	5.0	5.0	3.0	4.5	3.5	1.5	4.5	3.0	3.5	3.5	5.0	4.0	4.0	5.0	3.0	4.0	4.5	5.0	*Pib* , *Pit* , *Pa* , *Pii* , Unknown
131	659	Gie noi Ha Dong	Ha Tay	C	RL	I	II	A	3.5	3.0	3.5	3.0	1.0	3.0	5.0	4.5	4.5	5.0	4.5	5.0	3.5	1.0	5.0	5.0	5.0	4.0	5.0	4.5	5.0	3.0	4.5	4.0	3.5	5.0	*Pib* , *Pit* , Unknown
132	664	Gie tro Hoa Binh	Hoa Binh	NI	RL	—	II	A	5.0	2.0	3.0	3.0	1.0	5.0	5.0	4.5	4.0	4.0	3.0	5.0	3.0	1.0	5.0	3.0	4.0	4.5	4.0	5.0	5.0	5.0	5.0	4.0	4.5	5.0	*Pib* , *Pit* , Unknown
133	665	Gie Dong Thai Nguyen	Thai Nguyen	NI	RL	I	II	A	5.0	2.0	3.0	3.0	2.0	4.5	5.0	4.0	5.0	4.5	5.0	4.5	3.0	3.0	5.0	5.0	3.0	3.0	4.5	4.5	4.5	4.0	4.5	4.5	5.0	5.0	*Pib* , *Pit* , Unknown
134	666	Gie da Bac Ninh	Bac Ninh	RRD	RL	I	II	A	5.0	3.0	1.5	3.0	5.0	5.0	5.0	3.0	5.0	3.5	3.0	5.0	4.0	2.0	5.0	1.5	4.0	3.5	4.5	5.0	5.0	5.0	3.5	3.5	5.0	1.0	*Pib* , *Pit* , *Pi20* (t), Unknown
135	667	Gie nuoc Son Tay	Ha Tay	C	RL	I	II	A	4.5	3.0	2.0	3.0	1.0	3.5	4.5	5.0	4.0	5.0	1.5	5.0	3.0	1.0	5.0	5.0	4.5	4.0	5.0	5.0	5.0	5.0	5.0	5.0	5.0	4.5	*Pib* , *Pit* , Unknown
139	675	Gie do Nam Dinh	Nam Dinh	RRD	RL	I	II	A	4.5	3.0	3.5	3.0	1.0	5.0	5.0	5.0	5.0	5.0	5.0	4.5	3.0	2.0	4.0	5.0	5.0	5.0	5.0	3.0	3.5	4.5	4.5	5.0	5.0	5.0	*Pib* , *Pit* , Unknown
140	677	Gie mieng Tuyen Quang	Tuyen Quang	NI	UP	I	II	A	4.0	2.0	4.5	3.0	1.0	3.5	4.5	5.0	5.0	3.5	3.5	5.0	2.0	1.0	4.0	3.0	3.0	4.5	5.0	5.0	3.5	5.0	5.0	4.0	5.0	5.0	*Pib* , *Pii* , Unknown
142	680	Gie hoa Hai Duong(2)	Hai Duong	RRD	RL	I	II	A	5.0	3.0	1.5	3.0	1.0	3.5	5.0	5.0	4.5	5.0	3.0	5.0	2.0	1.0	5.0	2.0	5.0	3.5	5.0	5.0	5.0	5.0	3.0	5.0	5.0	5.0	*Pib* , *Pit* , *Pia, Pii,* Unknown
143	682	Gie nuoc Ninh Binh	Ninh Binh	RRD	RL	I	II	A	5.0	1.0	3.0	3.0	1.0	4.0	3.5	4.5	3.5	3.0	4.5	4.5	3.0	3.0	4.5	5.0	5.0	4.0	5.0	5.0	3.5	5.0	5.0	5.0	5.0	5.0	*Pib* , *Pit* , *Pik-s,* Unknown
144	683	Gie soc Nam Dinh	Nam Dinh	RRD	RL	I	II	A	4.5	2.0	4.5	3.0	1.0	4.5	5.0	4.5	4.0	5.0	5.0	5.0	3.0	1.0	4.5	5.0	5.0	4.5	5.0	5.0	5.0	3.5	4.0	5.0	5.0	5.0	*Pib* , Unknown
145	684	Gie ghenh Nam Dinh	Nam Dinh	Other	RL	I	—	A	5.0	1.0	3.0	3.0	1.0	5.0	3.0	5.0	5.0	4.5	3.0	5.0	3.0	1.0	4.5	3.0	5.0	5.0	4.5	3.5	5.0	5.0	4.5	4.5	4.5	5.0	*Pib* , *Pit* , Unknown
146	686	Gio Quang Binh	Quang Binh	C	RL	I	II	A	5.0	1.5	3.0	3.0	1.0	3.0	4.0	5.0	4.0	5.0	5.0	4.0	3.0	3.0	5.0	5.0	5.0	4.0	4.5	4.0	3.5	5.0	5.0	5.0	5.0	4.5	*Pib* , *Pit* , Unknown
149	738	Ba la Bac Can	Bac Cạn	NM	UP	I	II	A	4.5	2.0	3.0	3.0	1.0	3.0	3.5	3.5	4.0	5.0	4.5	4.5	3.0	3.0	5.0	5.0	4.0	4.5	4.0	4.0	4.5	3.0	4.5	4.0	5.0	5.0	*Pib* , *Pit* , Unknown
151	748	Du som Nam Dinh	Nam Dinh	RRD	RL	I	II	A	5.0	1.0	1.0	3.0	1.0	5.0	5.0	4.5	3.0	5.0	5.0	4.5	3.5	2.0	5.0	5.0	5.0	3.5	3.5	5.0	5.0	3.5	4.5	4.0	5.0	5.0	*Pib* , *Pit* , *Pik-s,* Unknown
152	750	Du thom Hai Phong*	Hai Phong	RRD	IL	J	II	A	3.0	3.0	1.0	3.0	1.5	3.0	3.5	4.0	4.5	5.0	5.0	4.0	3.5	3.0	5.0	2.0	2.0	5.0	5.0	3.0	4.5	4.5	3.0	4.5	4.5	5.0	*Pib* , *Pit* , *Pia,* One of *Pii* or *Pi5* (t), Unknown
157	780	Te hom Hung Yen	Hung Yen	RRD	RL	I	II	A	4.5	2.0	2.0	3.5	1.0	4.5	5.0	4.5	4.0	5.0	3.5	5.0	4.0	3.0	4.0	3.5	2.0	3.5	5.0	5.0	4.5	4.5	3.5	5.0	4.5	5.0	*Pib* , *Pit* , Unknown
160	787	Hom trang Son Tay	Ha Tay	C	RL	I	II	A	4.5	2.0	3.0	3.0	1.0	3.0	5.0	4.5	4.0	3.0	3.0	4.5	5.0	1.0	5.0	5.0	5.0	4.5	4.5	5.0	3.5	4.5	5.0	5.0	5.0	5.0	*Pib* , *Pit* , Unknown
161	788	Ech cau Hai Duong	Hai Duong	Other	RL	I	—	A	5.0	2.0	3.0	3.5	1.0	5.0	4.0	5.0	4.5	4.5	5.0	5.0	3.0	2.0	5.0	3.0	5.0	5.0	5.0	5.0	5.0	5.0	3.5	5.0	5.0	5.0	*Pib* , *Pit* , *Pia* , Unknown
163	800	Di do Hai Phong	Hai Phong	RRD	RL	I	II	A	5.0	1.0	1.0	3.5	1.0	3.0	3.5	4.5	5.0	5.0	3.0	4.5	2.0	2.0	5.0	3.0	5.0	3.5	4.5	5.0	5.0	3.5	4.5	3.0	5.0	5.0	*Pib* , *Pit* , *Pii* , Unknown
165	803	Dong trang Hai Phong	Hai Phong	Other	RL	I	—	A	4.0	1.0	3.0	3.0	1.0	3.5	3.0	4.5	3.5	5.0	3.0	3.5	3.0	2.0	4.0	5.0	2.5	4.5	3.5	4.5	5.0	4.5	5.0	3.5	4.5	4.0	*Pib* , *Pit* , *Pik-s* , Unknown
166	807	Hien gia loc Hai Duong	Hai Duong	RRD	RL	I	II	A	4.5	1.0	1.0	3.0	1.0	3.0	3.5	4.0	4.0	5.0	3.0	3.5	3.0	1.0	5.0	3.0	5.0	5.0	5.0	3.0	5.0	4.0	5.0	4.5	3.5	5.0	*Pib* , *Pit* , *Pii* , Unknown
168	869	Di dong Nam Dinh	Nam Dinh	RRD	RL	I	II	A	4.5	3.0	2.0	1.5	1.0	4.5	4.0	1.0	3.0	5.0	3.0	3.5	3.0	3.0	3.5	3.5	5.0	5.0	4.5	4.5	3.5	5.0	3.5	3.5	5.0	3.5	*Pib* , *Pia* , *Pi5* (t), *Pik-s* , Unknown
169	878	Hop Thanh Hoa	Thanh Hoa	C	RL	I	II	A	5.0	1.0	1.0	3.0	1.0	3.0	3.0	3.5	4.0	5.0	4.5	4.5	3.5	3.0	5.0	4.0	5.0	5.0	5.0	4.0	5.0	4.5	4.0	4.0	5.0	4.0	*Pib* , *Pit* , Unknown
190	3418	Lua ven dang 1	Quang Binh	Other	RL	J	—	A	3.5	1.0	3.0	2.0	4.0	5.0	3.5	1.0	3.0	3.0	2.0	3.5	5.0	5.0	3.5	3.0	5.0	3.5	3.5	3.5	3.5	3.0	3.0	3.0	5.0	1.0	*Pib* , *Pit*, *Pik-s* , *Pi20* (t), Unknown
194	7686	Ple lia	Thanh Hoa	C	RL	J	Ib	A	3.5	3.0	1.0	1.0	1.0	4.0	3.0	3.0	3.5	3.0	3.0	3.0	1.0	3.0	3.0	1.5	5.0	3.0	3.0	3.5	3.0	3.0	3.0	3.0	4.0	3.0	*Pia* , One of two, *Pi3* or *Pi5* (t), *Pik-s* , *Pi20* (t), Unknown

195	8222	Khau lech	Son La	NM	RL	J	Ib	A	3.0	3.0	3.0	1.5	2.0	3.0	3.0	3.0	5.0	3.0	3.0	3.0	3.0	1.0	3.0	1.0	2.0	4.0	5.0	5.0	3.0	4.5	1.5	4.5	3.0	3.0	*Pia* , one of three, *Pii* , *Pi3* , or *Pi5* (t), *Pi20* (t)
196	8225	Khau tan nhe	Son La	NM	UP	J	Ib	A	3.5	4.0	1.0	3.0	3.5	3.0	4.0	3.5	4.5	3.5	5.0	3.5	3.5	5.0	3.5	4.5	5.0	3.0	3.5	4.0	3.5	5.0	3.0	4.5	4.0	4.0	*Pit* , *Pia* , Unknown
200	9377	Beo Lang Son	Thanh Hoa	C	RL	I	II	A	3.0	1.0	2.0	3.0	3.5	5.0	3.0	3.5	3.0	3.5	1.0	3.0	4.0	5.0	3.5	5.0	5.0	3.0	3.0	3.5	1.5	2.0	5.0	3.0	3.0	3.0	*Pit* , *Pik-s* , *Pi20* (t), Unknown
204	BC15	BC15	Wide cultivation	Other	IL	I	II	A	3.0	1.0	1.0	4.0	3.0	5.0	3.5	1.0	3.5	5.0	1.0	4.5	4.5	4.0	5.0	5.0	5.0	4.0	3.0	5.0	3.5	4.0	3.0	3.0	4.0	2.0	*Pit* , *Pik-s* , *Pi20* (t), Unknown
3	35	Trung trang Tuyen Quang(1)	Tuyen Quang	NI	UP	I	II	B1	1.0	3.0	1.0	2.0	2.0	1.0	1.0	2.0	1.0	2.0	1.0	2.0	3.0	1.5	1.0	3.0	3.0	3.0	2.0	1.0	1.0	1.5	1.0	1.0	1.5	1.0	Unknown
28	235	Tam ruc Vinh Phuc	Vinh Phuc	RRD	RL	I	II	B1	1.5	1.0	2.0	1.5	4.0	1.0	2.0	1.5	1.5	1.0	1.0	1.5	4.5	5.0	1.5	1.0	3.0	1.0	3.0	1.5	2.0	1.0	2.0	1.0	1.5	1.0	*Pik-h* , *Pita-2*
34	260	Tam lap Kien An	Hai Phong	RRD	RL	I	II	B1	1.5	3.0	1.0	1.0	1.0	1.0	3.0	1.0	2.0	1.5	1.0	1.5	1.5	3.0	1.0	2.0	1.0	1.0	2.0	1.5	1.0	1.0	1.0	1.0	1.5	1.0	Unknown
35	261	Tam xoan Hai Duong*	Hai Duong	RRD	IL	I	II	B1	1.0	1.0	1.5	1.0	2.0	1.0	1.5	3.0	1.0	2.0	1.0	3.0	4.0	3.0	1.0	1.5	3.0	1.0	3.0	3.0	1.5	1.5	1.5	1.5	1.0	3.0	*Pik-m* or *Pik-h* , *Piz-t* , *Pita-2*
43	304	Tam thom Ninh Binh*	Ninh Binh	RRD	IL	J	II	B1	1.0	2.0	1.5	1.0	1.5	1.5	1.0	2.0	1.0	3.0	1.0	3.0	3.0	1.0	1.0	1.5	1.0	1.0	2.0	2.0	1.0	1.0	1.0	1.0	1.0	1.0	Unknown
44	305	Tam tron Hai Duong	Hai Duong	RRD	RL	J	II	B1	1.0	3.0	1.0	1.0	1.0	1.5	1.0	2.0	1.0	3.0	1.5	3.0	1.5	3.5	1.0	1.0	1.0	1.0	1.5	1.0	1.0	1.0	1.0	1.5	1.0	3.0	Unknown
49	315	Tam xoan	—	Other	IL	J	II	B1	1.0	1.0	1.0	1.0	1.0	1.0	1.0	1.0	1.0	1.5	0.0	3.0	1.5	5.0	1.0	2.0	1.0	1.0	1.5	1.0	1.0	1.0	1.0	1.0	2.0	3.0	Unknown
50	317	Tam xoan Vinh Phuc*	Vinh Phuc	RRD	IL	J	II	B1	1.0	1.5	1.0	1.0	1.5	1.0	1.0	1.5	1.0	2.0	1.0	3.0	3.0	1.0	1.0	1.5	1.0	1.5	3.0	1.0	1.0	1.0	1.0	1.0	1.0	3.0	Unknown
92	520	Doai Quang Binh	Quang Binh	C	RL	I	II	B1	2.0	1.0	1.0	1.5	1.0	2.0	3.0	2.0	1.0	2.0	1.0	2.0	3.0	3.0	3.0	1.0	1.0	3.0	3.0	3.0	2.0	3.0	3.0	3.0	2.0	1.5	Unknown
93	521	Nep rau Hoa Binh	Hoa Binh	NI	RL	I	II	B1	1.5	3.0	1.0	1.0	1.0	1.0	2.0	1.5	1.0	3.0	1.5	2.0	1.5	1.0	1.0	1.0	1.5	1.0	3.0	2.0	1.5	1.5	1.0	1.0	1.0	1.0	Unknown
97	527	Dau hen Thai Binh	Thai Binh	RRD	RL	I	II	B1	1.5	1.0	2.0	1.0	5.0	1.0	3.0	3.5	2.0	2.0	3.0	2.0	3.5	4.0	1.5	2.0	1.0	2.0	1.0	2.0	2.0	1.0	1.5	1.5	2.0	1.0	*Pib* , *Pit* , one of two, *Pik-h* or *Pik* , *Pita-2* , Unknown
100	534	Nep cai Nghe An	Nghe An	C	RL	I	II	B1	1.5	1.0	1.0	2.0	3.0	3.0	1.5	2.0	3.0	3.0	2.0	3.0	3.5	1.0	1.0	1.0	3.0	1.5	1.5	2.0	1.0	1.5	1.0	1.5	3.0	3.0	Unknown
101	543	Du Ninh Binh*	Ninh Binh	RRD	IL	I	II	B1	1.0	2.0	1.0	1.5	1.0	2.0	1.0	2.0	3.0	2.0	3.5	3.0	3.0	1.0	1.0	1.5	1.0	2.0	3.0	1.0	3.0	1.0	1.0	1.0	1.0	1.0	Unknown
119	607	Gie hoa Ha Tinh	Ha Tinh	C	RL	I	II	B1	2.0	1.0	1.0	1.5	1.0	1.0	1.5	1.0	2.0	1.0	1.0	2.0	3.5	3.0	1.0	1.0	3.0	1.0	2.0	1.5	3.0	3.5	1.0	1.5	3.0	3.0	Unknown
124	637	Gie doi Nam Dinh	Nam Dinh	RRD	RL	I	II	B1	1.0	2.0	1.0	3.0	1.0	1.0	1.0	1.0	1.0	1.5	2.0	3.0	3.0	1.0	2.0	1.5	1.5	1.0	1.0	2.0	1.0	1.5	1.0	1.0	1.0	1.0	Unknown
150	741	Lua luc Son Tay	Son Tay	RRD	RL	—	II	B1	2.0	2.0	1.5	1.0	1.0	3.0	2.0	2.0	1.0	3.5	1.0	1.5	3.0	3.0	1.0	1.0	1.0	1.0	3.0	1.5	3.0	1.5	1.5	1.0	2.0	3.0	Unknown
153	760	Trung trang Tuyen Quang	Tuyen Quang	NI	RL	I	II	B1	1.0	3.0	1.0	1.5	1.0	1.0	2.0	2.0	1.0	1.5	1.0	2.0	4.5	3.0	1.0	1.0	1.0	1.0	1.0	3.5	1.0	1.0	1.0	1.0	2.0	1.5	*Pish*
154	767	Bau đong Thanh Ha	Thanh Hoa	C	RL	I	II	B1	2.0	1.0	1.0	1.5	1.0	1.0	1.5	1.0	1.0	3.0	1.0	2.0	4.0	1.0	1.0	1.0	1.0	1.5	1.0	3.0	1.0	2.0	1.5	1.5	3.0	2.0	*Pish*
158	784	Hin ap be Nam Dinh	Nam Dinh	RRD	RL	I	II	B1	2.0	1.0	1.0	1.0	1.0	1.0	2.0	1.0	2.0	3.0	1.0	2.0	2.5	3.0	2.0	1.0	1.0	3.0	1.0	1.5	3.0	1.5	1.0	1.0	1.5	1.5	Unknown
159	785	Hop Hai Phong	Hai Phong	RRD	RL	-	II	B1	1.0	1.0	1.0	1.0	1.0	1.5	1.5	1.0	2.0	2.0	1.5	2.0	3.5	2.0	1.0	1.0	1.0	3.0	2.0	2.0	2.0	3.0	3.0	3.0	3.0	3.0	Unknown
162	799	Du trang Nam Dinh*	Nam Dinh	RRD	IL	—	II	B1	1.5	3.0	1.0	1.5	2.0	1.0	3.0	1.0	2.0	3.0	1.0	3.0	3.0	3.0	2.0	1.0	1.0	3.0	1.0	3.0	1.5	1.0	1.0	1.0	2.0	2.0	Unknown
164	802	Di trang Hai Duong	Hai Duong	RRD	RL	I	II	B1	4.0	1.0	1.0	2.0	1.0	1.0	1.5	1.0	1.0	2.0	3.0	3.0	3.0	1.0	1.5	1.5	1.5	1.0	1.0	3.0	1.5	1.5	1.0	1.5	2.0	1.0	*Pib* , One of three, *Pii* , *Pi3,* or *Pi19* (t), *Pik-h* , *Piz-t*
170	983	Te 3 thang	—	Other	RL	I	II	B1	1.0	1.0	1.5	1.0	1.0	1.0	2.0	1.0	1.0	1.0	1.0	2.0	3.0	3.0	2.0	1.5	2.0	3.0	1.0	1.5	1.0	1.0	1.0	1.0	2.0	2.0	Unknown
172	1181	Chiem Khau lo	—	Other	RL	J	II	B1	1.0	1.0	1.0	1.0	3.5	2.0	2.0	2.0	3.0	2.0	1.5	1.5	5.0	5.0	1.0	1.5	1.0	1.5	3.0	1.0	1.0	1.0	1.5	2.0	1.5	1.0	*Pik-h* , *Pita-2* , Unknown
173	1183	Chiem trang vo Hai Phong	Hai Phong	RRD	RL	J	II	B1	1.0	2.0	1.0	1.0	4.0	2.0	2.0	1.0	1.0	1.0	2.0	1.5	5.0	3.5	1.0	1.0	5.0	3.0	3.0	1.0	2.0	1.0	1.0	1.0	1.0	1.0	*Pi5* (t), *Pita-2* , Unknown
175	1252	Tam Chiem Ha Nam	Ha Nam	RRD	RL	J	II	B1	1.0	1.0	1.0	1.0	3.5	1.0	1.0	1.0	1.0	2.0	1.0	1.5	5.0	5.0	2.0	4.0	1.0	1.0	1.0	1.0	1.0	1.0	1.5	1.0	2.0	1.0	*Pi1* , *Pita-2* , Unknown
176	1266	Tep Nghe An	Nghe An	C	RL	J	II	B1	2.0	1.0	1.0	1.0	3.0	3.0	1.0	1.0	3.0	1.0	1.0	1.0	4.5	5.0	2.0	1.5	3.0	1.0	3.0	3.0	1.0	3.0	1.0	1.5	1.5	1.0	*Pish*
177	1269	Tep Hai Duong	Hai Duong	RRD	RL	J	II	B1	1.0	1.0	1.0	1.5	3.5	3.0	1.0	1.0	1.0	1.0	1.0	2.0	4.0	5.0	3.0	5.0	3.0	2.0	3.5	1.0	1.0	3.0	3.0	2.0	1.0	3.0	One of *Pi1* or *Pi7* (t), One of *Pita-2* and *Pi12* (t), Unknown
178	1278	Nep cai Chiem 2	—	Other	RL	J	II	B1	1.0	2.0	1.0	1.0	5.0	3.0	1.5	1.0	2.0	1.0	1.0	1.5	5.0	5.0	3.0	3.0	1.0	3.0	4.0	1.0	1.5	1.0	2.0	1.5	1.0	2.0	One of *Pi1* or *Pi7* (t), *Pita-2* , Unknown
183	2411	Tep 62	—	Other	RL	J	II	B1	1.0	1.0	1.0	1.0	3.5	3.0	1.0	1.0	2.0	1.0	1.0	1.5	3.5	5.0	1.0	1.5	3.0	1.5	3.0	1.0	1.0	1.0	1.0	1.5	1.5	1.5	*Pia* , *Pik-h* , *Pita-2*
184	2412	Te Tep*	—	Other	IL	J	—	B1	1.0	1.0	1.0	1.0	5.0	1.0	1.0	1.0	3.0	1.0	1.0	1.0	5.0	5.0	1.0	1.0	1.0	2.0	1.5	1.0	1.0	1.0	1.0	1.0	1.0	1.0	*Pik-h* , *Pita-2*
185	2431	Chiem ngan	—	Other	RL	J	II	B1	1.0	1.0	1.0	1.0	5.0	1.0	1.5	1.0	1.0	1.0	1.0	1.0	5.0	5.0	1.0	1.0	1.0	2.0	3.0	1.5	1.0	1.0	1.0	1.5	1.5	1.5	*Pik-h* , *Pita-2*
186	2432	Chiem nho Bac Ninh 2	—	RRD	RL	J	II	B1	1.5	1.0	1.5	1.5	4.5	3.0	3.0	1.5	1.0	1.0	2.0	1.0	4.5	5.0	3.0	1.0	3.0	1.0	3.0	1.0	2.0	1.0	1.0	1.0	1.0	1.5	*Pik-h* , *Pita-2*
187	2470	Nep Chiem	Sone La	NM	RL	J	II	B1	1.0	1.5	1.0	1.5	2.0	3.0	3.0	1.0	1.0	1.0	1.0	1.0	3.0	2.0	2.0	1.0	3.0	3.0	1.0	3.0	1.5	1.0	1.5	1.0	1.5	1.0	Unknown
188	2629	B'le la	Lai Chau	NM	UP	I	II	B1	1.0	1.0	1.0	1.0	3.0	1.0	3.0	1.5	1.5	1.5	1.0	2.0	5.0	5.0	3.0	5.0	3.0	2.0	2.0	3.0	3.0	1.5	1.0	1.0	1.5	3.0	One of two, *Pi1* or *Pi7* (t), *Pita-2* , Unknown
189	3345	Lua goc do	Quang Nam	C	UP	I	II	B1	1.0	1.0	1.0	3.0	4.0	1.0	1.0	1.0	1.0	1.0	1.0	1.5	3.5	4.0	2.0	1.0	1.0	1.0	1.0	1.0	1.0	1.0	1.0	1.0	1.0	1.0	*Pik-h* , *Pita-2* , Unknown
208	J01	J01	Wide cultivation	Other	IL	J	—	B1	1.0	1.0	1.0	1.0	5.0	1.5	1.0	1.0	1.5	1.0	1.0	3.0	3.0	5.0	1.0	1.0	1.0	1.0	4.0	1.0	1.0	1.0	1.0	1.0	1.0	1.0	One of two, *Pi1* or *Pik* , *Pita-2* , Unknown
5	46	Bat đen Thanh Hoa	Thanh Hoa	C	RL	I	II	B2	3.0	2.0	1.0	1.5	1.5	1.5	3.0	3.5	3.0	2.0	1.0	3.0	2.0	1.0	3.5	1.0	3.0	2.0	3.0	1.0	2.0	3.0	1.5	4.0	3.0	3.0	*Pib* , *Pi7* (t), *Piz-5* , unknown
8	52	Mo thu Bac Can	Bac Kan	NM	UP	J	II	B2	2.0	3.0	1.0	1.0	1.0	3.0	3.0	5.0	5.0	2.0	5.0	3.0	3.0	3.0	3.0	5.0	3.0	1.5	5.0	3.0	1.0	3.0	3.5	3.0	3.0	5.0	*Pia* , *Pi19* (t), Unknown
9	56	Som Bac Giang	Bac Giang	NI	RL	I	II	B2	2.0	3.0	1.5	1.5	1.0	2.0	3.0	4.5	5.0	3.0	3.0	3.5	5.0	3.0	3.0	3.5	4.0	2.0	5.0	3.5	2.0	3.5	4.0	2.0	3.5	5.0	*Pib* , *Pit* , *Pita* , Unknown
16	148	Loc mo Ha Tinh	Ha Tinh	C	RL	J	II	B2	3.0	1.0	1.0	3.0	1.0	3.0	3.5	4.5	4.5	3.0	1.0	3.0	3.0	3.0	4.5	2.0	3.5	1.0	4.5	3.0	3.0	4.5	3.0	3.5	3.5	3.0	*Pib* , *Pit* , *Pii* or *Pi3* , Unknown
18	154	Cham sai Tay Bac	Tay Bac	Other	UP	I	—	B2	2.0	1.0	3.0	3.5	1.0	3.0	3.5	3.0	3.5	3.0	1.0	3.0	3.0	3.0	3.5	3.0	3.0	5.0	5.0	4.0	3.0	3.5	5.0	3.5	3.5	4.5	*Pib* , *Pii* or *Pi5* (t), *Pik-s* , Unknown
27	231	Tam beo Bac Ninh	Bac Ninh	RRD	RL	I	II	B2	2.0	1.0	1.0	2.0	1.0	3.0	3.5	5.0	3.0	4.0	1.0	3.0	2.0	3.0	3.0	3.0	4.0	1.0	5.0	3.0	5.0	4.5	3.0	4.5	3.5	4.5	*Pib* , *Pit* , *Pii* or *Pi3* , *Pik-s* , Unknown
32	243	Tam lun Ha Dong	Ha Tay	RRD	RL	I	II	B2	3.0	1.0	3.0	3.0	1.0	3.0	3.0	5.0	5.0	2.0	2.0	3.5	3.0	3.0	3.0	2.0	3.0	3.5	5.0	4.0	1.5	4.0	4.5	2.0	4.5	5.0	*Pib* , *Pit* , *Pii,* Unknown
36	276	Tam xoan Vinh Phuc*	Vinh Phuc	RRD	IL	I	II	B2	3.5	2.0	1.0	1.0	1.0	3.5	1.5	4.5	3.0	4.0	1.0	3.0	3.5	2.0	1.0	3.0	1.0	3.0	3.5	3.0	4.5	3.0	3.0	3.5	3.0	3.0	*Pit* , *Pi*i or *Pi3* , *Piz* , *Pita* , Unknown
39	289	Tam thom Thanh Ha*	Thanh Hoa	C	IL	I	II	B2	2.0	3.0	2.0	2.0	3.0	2.0	2.0	5.0	5.0	2.0	1.5	4.0	3.0	1.0	1.0	1.5	1.0	1.0	4.5	2.0	2.0	2.0	3.0	1.5	3.0	5.0	*Pib* , *Pit* , *Pii* or *Pi3,* Unknown
40	291	Tam lun Vinh Phuc	Vinh Phuc	RRD	RL	I	II	B2	1.5	5.0	1.0	3.0	2.0	1.5	1.5	4.5	4.5	2.0	2.0	4.5	3.0	1.0	1.0	1.0	1.0	1.0	5.0	3.0	3.0	1.0	3.0	1.0	2.0	5.0	*Pit* , *Pii* or *Pi3,* Unknown

45	307	Tam dung Hai Duong	Hai Duong	RRD	RL	J	II	B2	3.0	3.0	3.0	1.5	3.0	2.0	3.0	4.5	3.5	3.0	2.0	5.0	4.5	1.0	1.0	1.5	3.0	1.5	5.0	3.0	1.5	5.0	5.0	3.0	5.0	5.0	*Pib* , *Pit* , *Pik-s* , Unknown
48	313	Tam xoan co râu Hai Duong	Hai Duong	RRD	RL	J	II	B2	5.0	1.5	3.0	1.0	3.0	5.0	3.0	3.0	1.0	2.0	3.0	3.0	1.0	2.0	1.5	1.5	1.0	3.0	3.0	1.0	3.0	3.5	1.0	3.0	3.0	2.0	*Pib* , *Pit* , *Pia, Pi5* (t), *Piz-t, Pi19* (t), Unknown
51	318	Tam nho Bac Ninh*	Bac Ninh	RRD	RL	I	II	B2	2.0	3.0	3.0	3.0	1.0	2.0	3.0	4.5	4.5	2.0	1.5	4.5	3.0	2.0	3.0	3.0	4.5	3.0	5.0	3.0	2.0	5.0	4.5	4.0	5.0	5.0	*Pib* , *Pit* , *Pii,* Unknown
52	319	Tam nho Vinh Phuc	Vinh Phuc	RRD	RL	J	II	B2	2.0	3.0	1.0	1.0	1.0	3.0	3.5	5.0	5.0	3.0	1.0	3.0	3.0	1.0	1.0	3.0	3.0	3.0	5.0	3.0	3.0	3.5	5.0	2.0	3.5	5.0	*Pib* , *Pit* , *Pii, Pita* , Unknown
54	322	Nep trac Hoa Binh	Hoa Binh	NI	RL	I	II	B2	3.0	2.0	1.0	1.0	1.5	2.0	3.0	4.0	3.0	2.0	3.0	3.5	3.0	3.0	3.0	3.0	3.0	2.0	4.0	1.5	2.0	3.0	4.0	1.5	1.0	4.5	*Pib* , *Pit* , *Pii, Pik-s,* One of three,*Pi12* (t), *Pita* , or *Pi19(* t), Unknown
55	323	Nep ga gay Hưng Yên	Hung Yen	RRD	RL	I	II	B2	2.0	3.0	4.0	1.0	1.0	3.5	3.0	5.0	5.0	2.0	3.0	4.0	1.5	3.0	3.0	4.5	3.0	2.0	4.5	3.0	3.5	3.5	3.0	1.5	3.0	5.0	*Pib* , *Pia,* Unknown
56	324	Nep khau non Hoa Binh	Hoa Binh	NI	RL	I	II	B2	1.0	2.0	1.5	1.5	3.0	2.0	1.0	4.5	5.0	5.0	3.5	4.5	4.0	3.0	1.0	3.0	1.0	1.5	5.0	1.0	1.0	2.0	1.5	1.0	1.5	3.0	*Pib* , *Pit* , Unknown
68	353	Nep xa Hoa Binh	Hoa Binh	NI	RL	I	II	B2	4.5	2.0	3.0	1.0	1.0	3.0	4.5	5.0	1.5	3.0	4.5	4.0	5.0	1.0	3.0	3.0	3.0	1.0	5.0	2.0	3.0	3.0	5.0	4.5	5.0	5.0	*Pib* , *Pit, Pik-s,* Unknown
69	354	Nep ca Hoa Binh	Hoa Binh	NI	RL	I	Ia	B2	3.0	1.0	1.0	3.5	1.0	3.0	4.5	1.0	1.0	3.5	3.0	3.5	3.0	1.0	3.0	1.0	5.0	4.0	1.5	5.0	3.0	5.0	1.0	4.0	3.0	2.0	*Pib* , *Pit* , *Pia* , one of two, *Pii* or *Pi5* (t), *Pik-s* , *Pi20* (t), Unknown
70	355	Nep me Hoa Binh	Hoa Binh	NI	RL	I	II	B2	1.0	1.0	3.0	1.0	5.0	1.5	3.0	4.0	4.5	5.0	4.5	5.0	4.5	5.0	1.0	5.0	4.5	3.0	3.5	3.0	1.5	2.0	1.0	3.0	1.5	3.0	*Pib* , *Pit* , Unknown
75	403	Toc tay Lao Cai	Lao Cai	NM	UP	I	II	B2	1.0	1.0	3.0	3.0	1.5	3.0	2.0	4.5	2.0	2.0	1.0	1.5	3.0	3.0	1.5	3.0	2.0	1.0	3.5	1.5	3.0	2.0	4.5	1.5	3.0	3.5	*Pib* , *Pit* , *Pii, Pik-s,* One of three,*Pi12* (t), *Pita* , or *Pi19(* t), Unknown
81	418	Nep chanh Ha Dong	Ha Tay	RRD	RL	I	II	B2	2.0	2.0	3.0	3.0	3.0	3.0	3.5	5.0	3.0	1.0	3.0	3.0	4.5	3.0	3.0	3.0	3.0	2.0	3.0	3.0	1.0	4.0	5.0	3.0	4.5	4.0	*Pib* , *Pit* , *Pik-s* , *Pita* , Unknown
88	512	Mo trang Vinh Phuc	Vinh Phuc	RRD	RL	I	II	B2	3.5	1.0	1.5	3.0	1.0	5.0	5.0	4.5	4.0	1.5	3.0	3.0	3.5	3.0	3.0	4.0	3.0	3.0	5.0	3.0	3.0	5.0	4.5	3.5	5.0	5.0	*Pib* , *Pit* , Unknown
91	517	Xe tat Lai Chau	Lai Chau	NM	UP	I	II	B2	2.0	1.0	1.5	1.5	1.0	2.0	3.0	3.0	1.0	1.0	1.0	3.0	3.0	1.0	3.0	1.0	3.0	4.5	3.0	3.0	3.0	1.0	5.0	1.5	3.0	1.5	*Pib* , *Pit* ,One of two, *Pii* or *Pi5* (t), *Pik-s* , Piz-t, One of three, *Pi12* (t), *Pi19* (t), or *Pi20* (t), Unknown
94	523	Bang som Nghe An	Nghe An	C	RL	I	II	B2	3.5	3.0	1.0	3.0	1.0	3.5	3.0	3.5	1.5	3.0	3.0	2.0	3.0	3.0	5.0	1.0	4.0	3.0	2.0	3.0	3.5	2.0	3.0	3.0	5.0	3.0	*Pib* , *Pit* , *Pia* , One of three, *Pii, Pi3,* or *Pi5* (t), *Pik-s* , *Pita* , Unknown
95	524	Nep re Thai Binh	Thai Binh	RRD	RL	I	II	B2	3.0	2.0	1.0	3.0	1.0	3.0	2.0	3.0	2.0	2.0	1.5	2.0	3.0	1.0	3.0	1.5	5.0	2.0	1.0	3.0	3.0	2.0	3.5	3.0	5.0	3.0	*Pib* , *Pit* , *Pia* , One of three, *Pii, Pi3,* or *Pi5* (t), *Pik-s* , One of two, *Pita or Pi20* (t), Unknown
98	529	Te cay Hoa Binh	Hoa Binh	NI	RL	I	II	B2	3.0	1.0	1.5	2.0	1.0	3.5	3.0	1.0	3.0	1.0	4.5	3.5	3.0	1.5	1.0	4.5	3.0	1.0	3.0	1.5	2.0	3.0	3.0	1.0	3.5	2.0	*Pia* , *Pik-p* , *Pita-2* , Unknown
99	531	Nep mo Hoa Binh	Hoa Binh	NI	RL	I	II	B2	3.0	1.0	1.0	3.0	1.0	3.0	3.0	5.0	4.5	1.0	3.0	3.0	4.0	3.0	3.0	4.0	3.0	3.0	3.0	3.5	1.5	4.5	5.0	3.0	3.0	4.5	*Pib* , *Pit* , *Pita* , Unknown
103	547	Nep tien Ha Tinh	Ha Tinh	C	RL	I	II	B2	3.0	1.0	3.0	2.0	1.0	2.0	4.0	4.0	3.0	2.0	3.5	4.5	3.0	2.0	3.5	4.5	3.0	1.0	4.0	3.0	3.0	5.0	5.0	3.0	3.5	4.0	*Pib* , *Pit* , *Pik-s* , *Pi19* (t), Unknown
104	549	Dang di Son Tay	Ha Tay	C	RL	I	II	B2	1.5	1.0	2.0	1.5	1.0	1.0	1.5	5.0	5.0	5.0	4.5	5.0	3.0	3.0	3.0	3.5	1.0	3.0	4.5	3.5	1.0	3.0	2.0	3.0	3.0	5.0	*Pib* , *Pit* , *Pia* , One of two, *Pii* or *Pi3, Pi19* (t), Unknown
107	563	Nep muong phon Hoa Binh	Hoa Binh	NI	RL	I	II	B2	3.5	2.0	1.5	3.0	1.0	3.5	4.5	5.0	1.0	3.5	1.5	3.5	3.0	1.0	4.0	2.0	4.0	4.5	3.5	5.0	5.0	2.0	4.5	4.0	5.0	4.5	*Pib* , *Pit* , *Pii, Pik-s* , Unknown
108	564	Mua ray ruộng Hoa Binh	Hoa Binh	NI	RL	I	II	B2	3.0	1.0	1.0	2.0	2.0	3.0	2.0	5.0	3.0	2.0	4.0	3.5	3.0	3.0	3.5	3.0	4.5	3.0	5.0	3.0	3.0	3.5	4.0	3.0	5.0	4.0	*Pib* , *Pit* , *Pii, Pik-s* , Unknown
110	581	Ven thap Thanh Ha	Thanh Hoa	C	RL	I	II	B2	3.0	1.0	3.0	2.0	3.0	3.0	2.0	1.0	3.0	3.0	2.0	3.0	3.0	3.0	3.0	5.0	1.0	3.5	3.0	3.0	3.0	3.0	5.0	3.0	5.0	3.0	*Pib* , *Pit* , *Pik-s* , *Piz-t,* one of two, *Pita* or *Pi20* (t), Unknown
115	602	Gie tron Hai Duong	Hai Duong	RRD	RL	J	II	B2	2.0	3.0	3.0	1.5	1.5	1.0	3.0	5.0	4.0	3.0	3.0	3.5	3.0	1.0	2.0	4.5	3.0	1.5	4.5	3.0	1.5	3.5	3.5	1.0	3.5	5.0	*Pib* , *Pit* , *Pia* , One of *Pii or Pi3* , *Pita* , Unknown
126	641	Gie hien Nam Dinh	Nam Dinh	RRD	RL	I	II	B2	3.0	2.0	1.0	2.0	1.0	1.5	1.5	5.0	3.5	4.0	3.0	3.0	4.5	3.5	3.0	4.0	3.0	1.0	4.5	2.0	2.0	2.0	4.5	3.0	3.0	4.5	*Pib* , *Pit* , *Pk-s* , *Pita* , Unknown
127	643	Gie Thanh Ha(2)	Thanh Hoa	C	RL	I	II	B2	4.5	1.0	1.5	3.5	2.0	3.0	1.5	4.5	5.0	3.5	5.0	4.5	3.0	1.0	1.0	5.0	3.0	3.0	5.0	3.0	2.0	1.5	1.0	3.0	3.5	5.0	*Pib* , *Pit* , *Pa* , *Pi19* (t), Unknown
129	651	Gie do Thanh Ha	Thanh Hoa	C	RL	I	II	B2	3.0	3.0	1.5	1.0	3.0	2.0	1.5	3.0	3.0	3.5	3.5	3.5	3.5	3.0	2.0	2.0	3.0	2.0	4.5	3.0	1.5	3.0	3.0	3.0	3.0	1.0	*Pik-p* , *Piz-5* , *Pita-2*
130	656	Gie do Vinh Phuc	Vinh Phuc	RRD	RL	J	II	B2	3.0	2.0	1.0	1.0	3.0	2.0	3.0	3.5	1.5	1.0	2.0	1.5	4.0	3.0	1.5	3.0	3.0	3.0	5.0	4.0	3.0	3.5	3.0	1.0	1.5	3.5	*Pi5* (t), *Pita* -2, Unknown
136	671	Gie trang Thanh Ha	Thanh Hoa	C	RL		II	B2	3.0	3.0	1.0	1.0	1.0	2.0	4.0	5.0	5.0	3.0	3.0	3.0	4.5	3.0	3.5	5.0	3.0	3.0	5.0	3.0	2.0	3.0	5.0	4.0	4.5	4.5	*Pib* , *Pit* , Unknown
137	673	Gie bac Phu Tho	Phu Tho	NI	RL	J	II	B2	2.0	3.5	3.0	1.5	1.0	2.0	3.0	5.0	5.0	2.0	3.0	3.0	3.0	1.0	3.0	3.0	3.0	3.0	5.0	1.0	3.0	3.5	4.5	1.5	5.0	5.0	*Pib* , *Pit* , *Pii, Pita,* Unknown
138	674	Gie do Hoa Binh	Hoa Binh	NI	RL	I	II	B2	3.0	3.5	3.0	2.0	1.0	2.0	4.0	4.5	4.0	3.0	3.0	4.5	3.0	1.0	3.0	3.0	4.5	2.0	4.5	4.5	3.5	5.0	5.0	3.0	4.5	4.0	*Pib* , *Pit* , *Pii,* Unknown
141	679	Gie choi Thanh Ha	Thanh Hoa	C	RL	I	II	B2	3.0	1.0	1.0	1.5	1.0	1.0	4.0	5.0	3.0	3.0	2.0	3.0	2.0	2.0	3.0	3.0	3.5	1.0	5.0	3.5	1.0	5.0	4.5	3.0	5.0	4.5	*Pib* , *Pit* , *Pii, Pik-s,* Unknown
147	696	Gie Phu Tho	Phu Tho	NI	RL	I	II	B2	3.0	3.0	1.0	2.0	1.0	3.0	3.0	3.5	5.0	3.0	3.0	3.0	3.0	2.0	3.5	2.0	3.0	3.0	5.0	3.0	1.5	3.0	3.5	3.0	4.5	4.5	*Pib* , *Pit* , *Pia,* One of three, *Pii, Pi3* , or *Pi5* (t), *Pita,* Unknown
148	731	Chao dai Nghe An	Nghe An	C	RL	I	II	B2	1.5	1.5	1.0	3.0	1.0	1.5	3.0	3.0	3.0	3.0	3.0	2.0	3.0	2.0	3.0	1.5	1.5	3.0	3.5	3.5	3.0	3.5	5.0	3.0	4.0	5.0	*Pib* , *Pit* , *Pia,* One of two, *Pii* or *Pi5* (t), *Pik-s,* Unknown
155	773	Phong keo Ha Giang	Ha Giang	NM	UP	I	II	B2	2.0	1.0	1.5	1.0	2.0	1.0	3.0	4.0	3.5	3.5	3.0	4.5	4.0	3.0	1.0	5.0	1.0	1.5	3.5	2.0	2.0	2.0	3.0	1.5	3.0	5.0	*Pi7* (t), *Pi12* (t), Unknown
156	774	Nep cai Hai Phong	Hai Phong	RRD	RL	I	II	B2	3.0	2.0	1.0	1.0	1.0	1.0	3.0	5.0	3.0	3.0	3.0	4.0	3.5	3.0	1.0	3.0	1.0	2.0	4.5	3.0	2.0	1.0	2.0	1.0	3.0	5.0	*Pi7* (t), one of two, *Pii* or *Pi3* , *Piz* , Unknown
167	866	Du lun Hai Duong	Hai Duong	RRD	RL	I	II	B2	3.5	1.0	1.0	3.0	1.0	3.0	3.5	4.0	3.0	3.5	2.0	3.5	3.0	2.0	3.0	5.0	5.0	3.5	5.0	5.0	3.5	3.5	3.0	3.5	4.5	4.5	*Pib* , *Pit* , *Pia* , *Pik-s* , Unknown

171	1154	Chum quang 2-2	–	Other	RL	J	II	B2	1.0	3.0	1.0	1.0	3.0	1.0	3.0	3.0	1.5	1.5	1.0	2.0	3.5	5.0	1.0	1.0	1.0	2.0	4.5	3.0	1.0	1.0	3.5	2.0	2.0	1.0	*Pia* , One of two, *Pi1* or *Pik* , *Pita-2* , Unknown
174	1235	Sai duong 1	–	Other	RL	J	II	B2	3.0	1.5	1.5	3.0	3.0	2.0	1.5	3.0	2.0	2.0	1.0	3.0	5.0	4.0	3.0	4.0	3.0	3.0	3.0	5.0	3.0	1.0	2.0	1.0	4.0	1.0	*Pib, Pit* , *Pik-s* , *Pi20* (t), Unknown
179	1314	Khau con	Tay Bac	NM	UP	J	Ia	B2	3.5	1.5	3.0	3.0	2.0	1.0	3.5	2.0	3.0	3.0	2.0	3.0	4.0	1.5	5.0	5.0	2.0	3.5	3.0	3.5	3.0	1.0	3.0	1.0	4.0	3.0	One of *Pib* or *Pi20* (t), *Piz-t,* Unknown
191	4855	Ble tu	Hoa Binh	NI	RL	I	Ib	B2	1.5	3.0	2.0	2.0	2.0	2.0	1.5	3.0	3.0	3.0	2.0	2.0	3.0	4.0	3.0	3.0	2.0	1.0	3.0	3.0	1.5	3.0	3.0	3.0	2.0	3.5	*Pia* , *Pik-h* , *Pita-2*
193	7095	Khau hang khoai	Hoa Binh	NI	RL	J	Ib	B2	1.5	3.0	2.0	2.0	1.5	1.0	2.0	3.5	3.0	3.0	2.0	3.0	3.0	2.0	1.0	3.0	2.0	1.0	3.0	3.0	1.0	2.0	3.0	1.5	2.0	3.0	Unknown
197	8235	Khau hut danh	Son La	NM	UP	J	Ib	B2	3.0	1.5	1.0	1.5	1.0	1.0	2.0	3.0	2.0	3.0	1.0	3.0	2.0	3.0	3.0	2.0	1.5	1.5	3.5	1.0	1.5	1.5	3.0	4.0	2.0	3.0	One of three, *Pi1* , *Pik-p* , or *Pi7* (t), *Piz-t* , *Pita*
198	8709	Ple cho	Son La	NM	UP	J	II	B2	3.0	3.0	1.0	3.0	1.5	5.0	3.0	3.5	3.0	4.0	2.0	4.0	4.0	3.0	4.0	2.0	5.0	3.0	5.0	5.0	3.0	3.0	3.0	2.0	3.5	3.0	*Pi5* (t), *Pik-s* , *Pi20* (t), Unknown
201	KD	Khang Dan	Wide cultivation	Other	IL	I	II	B2	3.0	1.0	1.0	3.0	1.0	4.0	3.0	1.0	3.0	4.0	3.0	3.5	1.0	1.5	3.0	1.0	5.0	3.5	2.0	3.5	3.0	3.0	1.0	3.0	1.0	1.0	*Pia* , One of two, *Pi3* or *Pi5* (t), *Pik-s* , *Pi20* (t), Unknown
202	Q5	Q5	Wide cultivation	Other	IL	I	II	B2	3.5	1.0	1.0	4.5	1.0	5.0	3.5	1.0	1.0	4.5	4.5	4.5	3.0	1.0	3.0	1.0	5.0	4.0	3.0	5.0	4.5	4.0	1.0	4.0	2.0	1.5	*Pit* , *Pi3* , *Pik-s* , *Pi20* (t), Unknown
203	BT7	BacThom7	Wide cultivation	Other	IL	I	II	B2	3.0	2.0	1.0	3.0	1.0	5.0	3.0	3.5	3.0	3.0	3.0	3.0	1.0	2.0	3.0	1.5	3.0	1.5	3.0	5.0	3.0	3.5	2.0	3.0	3.5	1.5	*Pi3* , *Piz-t* , *Pi12* (t), Unknown
205	Jas	Jasmine	Wide cultivation	Other	IL	I	II	B2	3.0	1.0	1.0	3.0	1.0	5.0	3.0	1.0	2.0	3.0	2.0	3.0	4.0	2.0	3.5	3.0	3.0	3.5	3.0	3.5	3.5	2.0	3.5	2.0	4.0	1.5	*Pia* , One of two, *Pi3* or *Pi5* (t), *Pik-s* , *Pi20* (t), Unknown
206	HT1	HT1	Wide cultivation	Other	IL	I	II	B2	3.0	1.0	1.0	3.0	3.0	4.5	3.0	1.5	3.5	3.0	1.0	3.0	1.0	1.0	3.0	1.0	3.5	1.5	2.0	4.0	4.0	3.0	1.0	3.0	3.5	1.0	*Pi3* , *Piz* , *Pi20* (t), Unknown
207	LT25	LT25	Wide cultivation	Other	IL	I	II	B2	1.0	1.0	1.0	1.5	1.0	1.0	1.5	1.0	3.5	4.0	1.0	3.5	1.0	1.0	3.0	1.0	4.0	2.0	2.0	3.0	2.0	3.0	1.0	3.0	2.0	1.0	*Pib* , *Pik-s* , *Piz-5* , *Pita* , Unknown
85	471	Nep thau dau Thai Binh	–	RRD	RL	–	Ia	–	–	–	–	–	–	–	–	–	–	–	–	–	–	–	–	–	–	–	–	–	–	–	–	–	–	–	–
96	526	Lua nuoc ruong Hoa Binh	–	NI	RL		II	–	–	–	–	–	–	–	–	–	–	–	–	–	–	–	–	–	–	–	–	–	–	–	–	–	–	–	–
180	1321	Ta vong	–	NM	RL	–	Ia	–	–	–	–	–	–	–	–	–	–	–	–	–	–	–	–	–	–	–	–	–	–	–	–	–	–	–	–
181	1453	Lua Nep xa va	–	other	RL	–	Ia	–	–	–	–	–	–	–	–	–	–	–	–	–	–	–	–	–	–	–	–	–	–	–	–	–	–	–	–
182	2173	Lua dai	–	C	RL	–	II	–	–	–	–	–	–	–	–	–	–	–	–	–	–	–	–	–	–	–	–	–	–	–	–	–	–	–	–
192	5791	Lua tai nguyen	Tra Vinh	S	RL	J	II	–	–	–	–	–	–	–	–	–	–	–	–	–	–	–	–	–	–	–	–	–	–	–	–	–	–	–	–
199	9209	Bat tam bang	Soc Trang	S	UP	J	II	–	–	–	–	–	–	–	–	–	–	–	–	–	–	–	–	–	–	–	–	–	–	–	–	–	–	–	–
209	J02	J02	Wide cultivation	Other	IL	J	–	–	–	–	–	–	–	–	–	–	–	–	–	–	–	–	–	–	–	–	–	–	–	–	–	–	–	–	–
210	8200	Kasalath	Control	Other	IL	I	Ib	A	4	1.5	1	3	1	5	3.5	4.5	1.5	5	2	4.5	3	2	4.5	5	5	3.5	4.5	4	5	5	5	5	4.5	5	*Pib* , *Pit* , *Pik-s* , Unknown
211	9048	Nipponbare	Control	Other	IL	J	Ia	B2	2	3	1	1	1	1	2	2	3.5	3	3	3	1	1	3	3	3	1.5	2	3	2	2	2	1	3	1.5	Unknown
212	–	US-2	Control	Other	IL	I	–	A	5	5	5	5	5	5	5	4	5	5	5	5	5	5	5	5	5	5	5	5	5	5	5	5	5	5	Non
213	–	LTH	Control	Other	IL	J	–	A	5	5	5	5	5	5	5	5	5	5	5	5	5	5	5	5	5	5	5	5	5	5	5	5	5	5	Non
Differential vareity's groups	"U"	ML*Pish-B*	Control	–	IL	J	–	–	R	R	R	R	S	R	R	R	R	R	R	R	S	R	R	R	R	R	R	R	R	R	R	R	R	R	*Pish* on chromosome 1
		ML*Pib-B*	Control	–	IL	J	–	–	S	R	S	S	S	S	S	S	S	S	S	S	S	S	S	S	S	S	S	S	S	S	S	S	S	S	*Pib* on chromosome 2
		ML*Pit-K59*	Control	–	IL	J	–	–	S	S	R	S	S	S	S	S	S	S	S	S	S	S	S	S	S	S	S	S	S	S	S	S	S	S	*Pit* on chromosome 1
		LTH	Control	–	IL	J	–	–	S	S	S	R	S	S	S	S	S	S	S	S	S	S	S	S	S	S	S	S	S	S	S	S	S	S	Non
		ML*Pia-A*	Control	–	IL	J	–	–	S	S	S	S	R	S	S	S	S	S	S	S	R	S	S	S	S	S	S	S	S	S	R	S	S	S	*Pia* on chromosome 11
	"i"	ML*Pii-F5*	Control	–	IL	J	–	–	S	S	S	R	R	R	S	S	S	S	S	S	R	R	S	R	S	S	S	S	S	S	S	S	S	S	*Pii* on chromosome 9
		ML*Pi3-CP4*	Control	–	IL	J	–	–	S	S	S	S	R	S	R	S	S	S	S	S	R	R	S	R	S	S	S	S	S	S	R	S	S	S	*Pi3* on chromosome 9
		ML*Pi5-M*	Control	–	IL	J	–	–	S	S	R	S	S	S	S	R	S	S	S	S	S	R	S	R	S	S	S	S	R	S	S	S	S	S	*Pi5* (t) on chromosome 9
	"k"	ML*Pik*^{s}*-F5*	Control	–	IL	J	–	–	S	S	S	S	S	S	S	S	R	S	S	S	S	S	S	S	S	S	S	S	S	S	S	S	S	S	*Pik-s* on chromosome 11
		ML*Pik*^{m}*-Ts*	Control	–	IL	J	–	–	S	S	R	R	S	R	R	R	R	R	S	S	S	S	R	R	R	R	R	R	R	R	R	R	S	R	*Pik-m* on chromosome 11
		ML*Pi1-CL*	Control	–	IL	J	–	–	S	S	R	R	S	R	R	R	R	S	R	S	S	S	R	S	R	R	S	R	R	R	R	S	R	R	*Pi1* on chromosome 11
		LthNIL*Pik*^{h}*-K3*	Control	–	IL	J	–	–	S	S	R	R	S	R	R	R	R	S	R	R	S	S	R	R	R	R	R	R	R	R	R	R	R	R	*Pik-h* on chromosome 11
		LthNIL*Pik-Ka*	Control	–	IL	J	–	–	S	S	R	R	S	R	R	R	S	S	S	S	R	S	R	R	R	R	S	R	R	R	R	R	R	R	*Pik* on chromosome 11
		ML*Pik*^{p}*-K60*	Control	–	IL	J	–	–	S	S	S	R	R	R	R	R	S	S	S	S	S	R	S	S	R	R	S	R	R	R	S	S	R	S	*Pik-p* on chromosome 11
		ML*Pi7-M*	Control	–	IL	J	–	–	S	S	R	R	S	R	R	S	R	S	S	S	S	S	R	S	R	R	S	R	R	R	R	S	R	S	*Pi7* (t) on chromosome 11
	"z"	ML*Pi9-W*	Control	–	IL	J	–	–	R	R	R	R	R	R	R	R	S	S	R	S	R	R	S	R	S	R	R	R	R	S	R	R	R	R	*Pi9* (t) on chromosome 6
		ML*Piz-Fu*	Control	–	IL	J	–	–	S	R	S	S	R	S	S	S	R	S	R	S	R	R	R	S	R	S	S	S	S	S	R	S	R	S	*Piz* on chromosome 6
		ML*Piz*^{5}*-CA*	Control	–	IL	J	–	–	S	S	R	R	S	R	R	R	S	S	R	S	R	R	S	R	S	R	S	R	R	R	R	S	R	R	*Piz-5* on chromosome 6
		ML*Piz*^{t}*-T*	Control	–	IL	J	–	–	S	S	R	S	R	S	S	R	R	R	R	S	S	R	S	S	R	S	R	S	S	R	S	S	S	R	*Piz-t* on chromosome 6
	"ta"	ML*Pita*^{2}*-Pi*	Control	–	IL	J	–	–	R	R	R	R	S	R	R	R	R	R	R	R	S	S	R	R	R	R	S	R	R	R	R	R	R	R	*Pita-2* (Pi) on chromosome 12
		ML*Pita*^{2}*-Re*	Control	–	IL	J	–	–	R	R	R	R	S	R	R	R	R	R	S	R	S	S	R	S	S	R	S	S	R	S	R	R	R	R	*Pita-2* (Re) on chromosome 12
		ML*Pi12-M*	Control	–	IL	J	–	–	S	R	R	S	S	S	S	S	S	S	S	S	S	S	S	S	R	S	R	S	S	R	S	S	R	S	*Pi12* (t) on chromosome 12
		ML*Pita-K1*	Control	–	IL	J	–	–	R	R	S	S	R	R	R	R	S	R	S	R	R	R	R	R	R	R	S	R	S	R	R	S	R	S	*Pita* (K1) on chromosome 12
		ML*Pita-CP1*	Control	–	IL	J	–	–	R	S	S	R	R	R	R	S	S	S	R	R	S	R	R	S	S	R	S	R	S	S	S	R	S	S	*Pita* (CP1) on chromosome 12
		ML*Pi19-A*	Control	–	IL	J	–	–	S	S	R	S	R	S	S	S	S	S	S	S	R	R	S	S	R	S	S	R	S	S	S	S	R	S	*Pi19* (t) on chromosome 12

		ML*Pi20-IR24*	Control	–	IL	J	–	–	S	R	S	S	S	S	S	R	S	S	S	S	S	S	S	S	S	S	S	S	S	S	S	S	S	R	*Pi20* (t) on chromosome 12

International differential variety's set was consisted of 23 monogentic lines (Tsunematsu et al., 2000) and two LTH NILs (Telabanco-Yanoria et al., 2010)

Rice accessions were categoeized into Indica and Japonica Groups by Plant Resources Center, Vietnam

NM: Mountanous area in Nort region, NI: Intermediate area between mountanous and Red River Delta areas in North region, RRD: Red River Delta area in North region, C: Central region, S: South region.

J: Japonica Group, I: Indica Group

IL: Irrigated lowland, RL: Rainfed lowland, IL/RL: Borth use for IL and RL, UP: Upland

Rice accessions were classfied into three cluster groups, Ia, Ib, and II, based on 65 polymorphisum data of 14 SSR markers.

Rice accessions were classified into three cluster groups, A, B1, and B2, based on rectionpatterns of 26 standard differntial blast isolates by Nguyen et al., 2020)

The evaluation of resistance were performed using six scores (0-5), and scors: 0.0-2.9, were categorized into resistant(R), and 3.0-5.0 were susceptible (S).

* High eating quality cultivars

Supplemental Table 2. SSR markers for polymorphism analysis used in this study

Entry No.	Chr.	Locus Name	Map position (cM)	Forward Primer	Reverse Primer	No. of alleles detected
1	1	RM495	0.3	AATCCAAGGTGCAGAGATGG	CAACGATGACGAACACAACC	3
2	1	RM3604	26.8	ATGTCAGACTCCGATCTGGG	TCTTGACCTTACCACCAGGC	5
3	1	RM1	29.7	GCGAAAACACAATGCAAAAA	GCGTTGGTTGGACCTGAC	6
**4**	**1**	**RM8111**	**30.8**	**AGGTAACTAAGCTAGGTGTT**	**TAGGTACAGTAATACCAAGC**	**4**
**5**	**1**	**RM259**	**38.8**	**TGGAGTTTGAGAGGAGGG**	**CTTGTTGCATGGTGCCATGT**	**3**
6	1	RM3359	42.4	ACTCCGCTAATCACCACCAC	GAGGAGGAGAGGGAAAGGAG	8
**7**	**1**	**RM6840**	**181.1**	**TACCAAGACTCCGCTATGGC**	**GAAGAAGGGATCATGGATCG**	**5**
8	1	RM8137	181.1	GTAATTGAATTTCACTGCTGCT	ACGTACGTGACGTGCTTATG	5
9	2	RM3865	21.1	AACCATGGACAGTTGAACAC	CTCCGACAAGAACTTCCTC	4
10	2	RM1347	26.6	AACAAATTAAACTGCCAAG	GTCTTATCATCAGAACTGGA	6
11	2	RM6378	28	ATAGGGTGGGTGTGCTGAAC	TGCACAAAACTGCAGGTCTC	7
12	2	RM324	66	CTGATTCCACACACTTGTGC	GATTCCACGTCAGGATCTTC	5
13	2	RM262	81.4	CATTCCGTCTCGGCTCAACT	CAGAGCAAGGTGGCTTGC	**4**
14	2	RM3874	94.3	TGGGTGATCTTAGTTTGGCC	AATGTGCCTGCACATGTCAC	7
15	2	RM1367	110.9	GCATCGTTCATGTACACTGG	CTGCTACGCTGCTACTCCTAG	7
**16**	**2**	**RM240**	**135.5**	**CCTTAATGGGTAGTGTGCAC**	**TGTAACCATTCCTTCCATCC**	**6**
**17**	**2**	**RM406**	**156.3**	**GAGGGAGAAAGGTGGACATG**	**TGTGCTCCTTGGGAAGAAAG**	**3**
**18**	**3**	**RM5474**	**18.6**	**AAAGTGTTGGTGAGCATAGC**	**TTTGTGTTTGGAGAGACGAG**	**5**
19	3	RM6959	65.4	TCCTATGGAGGATTGTTGCC	CGGAGGAGCAGAACAAAAAC	5
20	3	RM8208	89	GCCCAAACTACACTCTCTTG	GTAAATGCCTGAGTGCCTAC	5
21	3	RM168	122.8	TCCTCCATCTCCTCCGCTCCCG	GGGCGTGGTGGCCTTCTTCGTC	3
22	3	RM8203	140.1	CATTGATAATGTCCAGTGACG	CTCCTGTTGTCATTCTTTGG	4
23	3	RM7000	152.2	CCCTTCTTTTCAACTGAATA	TTGTAACAATGAACTCGTTC	9
24	3	RM7389	164.4	AGCGACGGATGCATGATC	TTGAGCCGGAGGTAGTCTTG	3
25	4	RM8213	10.7	AGCCCAGTGATACAAAGATG	GCGAGGAGATACCAAGAAAG	5
**26**	**4**	**RM3317A**	**25.4**	**CCTGACAGAAGAATGGTACACC**	**TGTGGCTTCTCGTTGAGTTG**	**4**
27	4	RM5586	56.1	CTCCATAATCAAGGAAGCTA	ATGAGTTCTTTCGTCAGTGT	4
**28**	**4**	**RM3524**	**68.3**	**CGGAGCTGGTCTAGCCATC**	**GTCTCCGTCTTCCTCACTCG**	**5**
29	4	RM3367	74.5	GGATCCATCCATCCACTGAC	GGATATGTGCTGCTGTGTGC	6
30	4	RM3836	108.2	ACTGTGGAGTACAGGTCGGC	GAAACGGAAACGAAACCCTC	3
31	5	RM405	24.7	TCACACACTGACAGTCTGAC	AATGTGGCACGTGAGGTAAG	2
32	5	RM267	25	TGCAGACATAGAGAAGGAAGTG	AGCAACAGCACAACTTGATG	3
33	5	RM413	26.7	GGCGATTCTTGGATGAAGAG	TCCCCACCAATCTTGTCTTC	4
34	5	RM1089	37.2	CAGAAGGATTATCTCGATACC	AATAGGGCTTGAAATAAATTG	5
35	5	RM3663	87.4	CATCAACCTCCACGAACATG	CTCGGTGGTGATCCTCCTC	4
36	5	RM3476	101	GATTCTCGTCGTAATCAAGA	ATCCACGGTTAAGATAAATG	6
37	5	RM3790	107.7	TAATTGCGGTCTCGTGCC	AACCACCTCAACTACTGCCG	5
38	5	RM6313	116.5	ATCCAGATCCACTTTGACCG	GGAGGACTTCTACCATCCTTG	2
39	6	RM508	2.3	GGATAGATCATGTGTGGGGG	ACCCGTGAACCACAAAGAAC	2
40	6	RM510	11.5	AACCGGATTAGTTTCTCGCC	TGAGGACGACGAGCAGATTC	3
41	6	RM276	30.8	CTCAACGTTGACACCTCGTG	TCCTCCATCGAGCAGTATCA	5
**42**	**6**	**RM8225**	**54.1**	**TGTTGCATATGGTGCTATTTGA**	**GATACGGCTTCTAGGCCAAA**	**4**
43	6	RM162	108.3	GCCAGCAAAACCAGGGATCCGG	CAAGGTCTTGTGCGGCTTGCGG	4
44	6	RM3138	110.6	TTGACAAGAGATCAAGGCGG	GTGAATGTTGAGCTGCATGG	5
45	7	RM3831	5.2	CTCCACGTTCTCCGACGAG	GCGGCAACTCCTACATATCC	4
46	7	RM1134	25.4	ACACCCAACTTTTCTCACGC	AGCTAGGGTTTCGATCTCCC	2
47	7	RM7121	41.7	GGAGATGGCACACGTCAAAC	AGGATCCCGTTTTGTAGCAG	2
48	7	RM11	67	TCTCCTCTTCCCCCGATC	ATAGCGGGCGAGGCTTAG	3
49	7	RM234	93.9	ACAGTATCCAAGGCCCTGG	CACGTGAGACAAAGACGGAG	4
**50**	**7**	**RM8261**	**95.5**	**GACGACTGGATGGTACGAC**	**TGCTTCTCCTGCAAACAC**	**4**
51	8	RM152	3.3	GAAACCACCACACCTCACCG	CCGTAGACCTTCTTGAAGTAG	4
**52**	**8**	**RM3395**	**52.9**	**ACCTCATGTCCAGGTGGAAG**	**AGATTAGTGCCATGGCAAGG**	**5**
53	8	RM330	60.9	CAATGAAGTGGATCTCGGAG	CATCAATCAGCGAAGGTCC	2
54	8	RM3153	66.5	CGGTTCTTTTCACATGGTCG	ATCACAACAAGCTCGACGTG	5
55	8	RM284	78.5	ATCTCTGATACTCCATCCATCC	CCTGTACGTTGATCCGAAGC	2
56	8	RM7356	78.5	CCAAGGACACATATGCATGC	GCAATTCATGGCGCTGTTC	5
57	8	RM6948	114.4	GGTAAGTTGTCGGTTGCCTC	ACGTCCATACCAGGTCAAGC	3
58	9	RM1328	0	CCATGAGTGACATCAAAAGG	CCATGAGTGACATCAAAAGG	3
59	9	RM3912	34.9	TGTGTGTGCCCGATCTAC	CCTCTCGATGAGCATTCC	2
60	9	RM6051	42.5	AGGCTGATCCAAGATCCATG	CCCGGAGGCTGATTCTTG	3
61	9	RM3700	55.3	AAATGCCCCATGCACAAC	AAATGCCCCATGCACAAC	3
**62**	**9**	**RM7048**	**62.4**	**CAACCCCTAATTTCACGCTC**	**GACTTCACTGGCACTGGATG**	**4**
63	9	RM3164	72.1	TCCTCCTGCTAGCTGCCTAG	TCGCCTTCCTTTTCACTCAC	5
64	10	RM8201	26.1	TCTGTTTATAAGCGCAGCAC	GCCGGCGAGCTACTACTAC	2
65	10	RM6370	1.1	TTGACAAGCCACACACACAG	GTCCTCCCTTGGTTCTTTCC	3
**66**	**10**	**RM258**	**48.8**	**TGCTGTATGTAGCTCGCACC**	**TGGCCTTTAAAGCTGTCGC**	**5**
67	10	RM171	55.59	AACGCGAGGACACGTACTTAC	ACGAGATACGTACGCCTTTG	2
68	10	RM271	59.4	TCAGATCTACAATTCCATCC	TCGGTGAGACCTAGAGAGCC	3
69	10	RM6745	-	TGTTCTCAACACAAAATTCTCT	ATCGTAAGCAAAGTGCATAA	9
70	11	RM3133	-	TCAATAGACACACGGGCATG	CGATTTTGCTCACTGCACAG	4
71	11	RM552	40.6	CGCAGTTGTGGATTTCAGTG	TGCTCAACGTTTGACTGTCC	5
72	11	RM21	85.7	ACAGTATTCCGTAGGCACGG	GCTCCATGAGGGTGGTAGAG	5
73	12	RM1337	-	GTGCAATGCTGAGGAGTATC	CTGAGAATCTGGAGTGCTTG	7
74	12	RM247	26.7	TAGTGCCGATCGATGTAACG	CATATGGTTTTGACAAAGCG	4
**75**	**12**	**RM7619**	**38.1**	**CTTGGTATGTATTGGCAGCG**	**GAGGCAATAGGAGGGGAGAG**	**4**
76	12	RM7376	89.5	TCACCGTCACCTCTTAAGTC	GGTGGTTGTGTTCTGTTTGG	4
77	12	RM17	107.4	TGCCCTGTTATTTTCTTCTCTC	GGTGATCCTTTCCCATTTCA	5
78	12	RM1227	109.2	CATGGTAGCACACACCCTTG	CATCGACATGTGGACCACTC	3
79	12	RM1015	-	TGTATGACTTTTTAGCATTG	CCACATTCATTTAGATGTTA	3
Total (All markers)						336
Mean (All markers)						4.3
**Total (14 markers used for cluster analysis)**						65
**Mean (14 markers used for cluster analysis)**						4.6

A total of 79 SSR markers were used for polymorphism analysis. Among them, 14 SSR markers (Bold) which were able to collect the data completely from the whole rice accessions, were used for the cluster analysis for classification.
